# Canadian COVID-19 host genetics cohort replicates known severity associations

**DOI:** 10.1371/journal.pgen.1011192

**Published:** 2024-03-22

**Authors:** Elika Garg, Paola Arguello-Pascualli, Olga Vishnyakova, Anat R. Halevy, Samantha Yoo, Jennifer D. Brooks, Shelley B. Bull, France Gagnon, Celia M. T. Greenwood, Rayjean J. Hung, Jerald F. Lawless, Jordan Lerner-Ellis, Jessica K. Dennis, Rohan J. S. Abraham, Jean-Michel Garant, Bhooma Thiruvahindrapuram, Steven J. M. Jones, CGEn HostSeq Initiative, Lisa J. Strug, Andrew D. Paterson, Lei Sun, Lloyd T. Elliott

**Affiliations:** 1 Department of Statistics and Actuarial Science, Simon Fraser University, Vancouver, British Columbia, Canada; 2 Genetics and Genome Biology Program, The Hospital for Sick Children, Toronto, Ontario, Canada; 3 BC Children’s Hospital Research Institute, Vancouver, British Columbia, Canada; 4 Department of Medical Genetics, University of British Columbia, Vancouver, British Columbia, Canada; 5 Canada’s Michael Smith Genome Sciences Centre, BC Cancer Agency, Vancouver, British Columbia, Canada; 6 School of Epidemiology and Public Health, University of Ottawa, Ottawa, Ontario, Canada; 7 Dalla Lana School of Public Health, University of Toronto, Toronto, Ontario, Canada; 8 Lunenfeld-Tanenbaum Research Institute, Sinai Health, Toronto, Ontario, Canada; 9 Gerald Bronfman Department of Oncology, Department of Epidemiology, Biostatistics and Occupational Health, Department of Human Genetics, McGill University, Montreal, Quebec, Canada; 10 Lady Davis Institute for Medical Research, Jewish General Hospital, Montreal, Quebec, Canada; 11 Department of Statistics and Actuarial Science, University of Waterloo, Waterloo, Ontario, Canada; 12 Mount Sinai Hospital, Toronto, Ontario, Canada; 13 Department of Laboratory Medicine and Pathobiology, University of Toronto, Toronto, Ontario, Canada; 14 Department of Statistical Sciences, University of Toronto, Toronto, Ontario, Canada; University of Pennsylvania, UNITED STATES

## Abstract

The HostSeq initiative recruited 10,059 Canadians infected with SARS-CoV-2 between March 2020 and March 2023, obtained clinical information on their disease experience and whole genome sequenced (WGS) their DNA. We analyzed the WGS data for genetic contributors to severe COVID-19 (considering 3,499 hospitalized cases and 4,975 non-hospitalized after quality control). We investigated the evidence for replication of loci reported by the International Host Genetics Initiative (HGI); analyzed the X chromosome; conducted rare variant gene-based analysis and polygenic risk score testing. Population stratification was adjusted for using meta-analysis across ancestry groups. We replicated two loci identified by the HGI for COVID-19 severity: the *LZTFL1/SLC6A20* locus on chromosome 3 and the *FOXP4* locus on chromosome 6 (the latter with a variant significant at P < 5E-8). We found novel significant associations with *MRAS* and *WDR89* in gene-based analyses, and constructed a polygenic risk score that explained 1.01% of the variance in severe COVID-19. This study provides independent evidence confirming the robustness of previously identified COVID-19 severity loci by the HGI and identifies novel genes for further investigation.

## Author summary

Host genetics determine how human genetics contribute to the response to infectious disease. HostSeq is a Canada-wide effort to contribute to our understanding of host genetics for COVID-19. The HostSeq study involves genetic and clinical data from individuals who tested positive for SARS-CoV-2 across Canada. This work examines locations in the human genome that have been reported to be involved in severe COVID-19 worldwide and determines if we also see the involvement of those genomic locations in Canadians. This work also explores several ‘genetic quantities’ such as a determination of how much of COVID-19 severity is due to genetics in Canada.

## Introduction

The HostSeq project was initiated as a Canadian response to the global COVID-19 pandemic in April 2020 by CGEn (Canada’s national platform for genome sequencing and analysis). In brief, HostSeq assembled a databank based on 15 clinical and epidemiological studies with DNA samples and clinical information from ~10,000 Canadians infected by the SARS-CoV-2 virus. We have described the HostSeq resource in detail in previous work [1]. In this paper, we present genetic analyses of N = 8,474 of 10,059 joint-called HostSeq genomes that passed our extended quality control measures. Our primary outcome variable is hospitalization due to COVID-19.

There has been a long history of human genetic studies of susceptibility and severity of infectious diseases [2,3]. In the last 15 years, genome-wide association studies (GWAS) have identified numerous variants associated with complex human diseases or traits [4]. Variants associated with susceptibility to or severity of infectious diseases have provided insight into the genes and mechanisms involved. Loci identified from GWAS can be combined into a single score that reflects some of the genetic contributions to a complex trait, often called a polygenic risk score (PRS) [5]. Here we aim to conduct a GWAS and a PRS analysis for severe COVID-19, defined as being hospitalized after infection with SARS-CoV-2, using the CGEn HostSeq resource. Our hospitalization outcome maps to the ‘B1’ variable of the Host Genetics Initiative (HGI) [6], a global working group dedicated to COVID-19 host genetics [7].

Given our relatively small sample size (N = 8,474) compared to the much larger HGI meta-analyses (over 85,000 individuals included in the B1 contrast), we did not expect to have novel GWAS findings. Therefore, besides conducting a whole-genome scan in these Canadian data, we sought to replicate top associations reported by HGI and evaluate consistency between the two studies. As some HostSeq samples were part of the HGI meta-analysis (‘CGEN’ and ‘BQC19’; 8), we created a version of HGI results (referred to as ‘HGI7no’) by removing the effect of overlapping samples; see Methods for details. In HGI7no, there were three genome-wide significant loci (chr3:45805277, chr6:41515629, chr21:33249643), of which two were replicated in the HostSeq GWAS. The *FOXP4* locus on chromosome 6 passed genome-wide significance in our primary analysis. Additionally, a PRS constructed from these three loci was significantly associated with hospitalization status.

We also conducted post-GWAS analyses, including an ancestry stratified meta-analysis (SAIGE and MR-MEGA) [9,10]. We examined genotype-by-sex interactions (G x Sex) since there is strong epidemiological evidence that males are at increased risk for severe COVID-19 [11]. We performed functional mapping analysis on our primary GWAS results using *FUMA GWAS* [12], finding a significant association with *MRAS*. We analyzed gene-based coding variants using the optimal sequence kernel association test (SKAT-O) [13] to include rare variants in our analysis, finding a significant association with *WDR89*. Finally, we performed a SNP-based heritability analysis, using the linkage-disequilibrium score regression approach (LDSC) [14], and compared the HostSeq heritability estimate to that of HGI.

### Related work

While COVID-19 is caused by infection with the SARS-CoV-2 virus, work since the beginning of the pandemic has shown that human genetic variation modulates both susceptibility to infection and the severity of COVID-19. The current version of HGI, released in April 2022, found 51 loci across all phenotypic contrasts [8]. Of the 51 loci, 38 have been reported by earlier studies [15–20].

Variants identified in these COVID-19 GWAS are linked to viral entry into host cells (*SLC6A20*, *SFTPD*, and *TMPRSS2*), the type I interferon pathway (*IFNAR2*, *TYK2*, *JAK1*, *IRF1*, *IFNA10*, *TLR7*, and *DOCK2*), the inflammatory pathway (*OAS* gene cluster, *DPP9* and *TYK2*), as well as lung function and respiratory diseases (such as *MUC5B*, *DPP9*, and *FOXP4*). The strongest and most consistent finding for COVID-19 severity is the 3p21.31 region containing multiple protein-coding genes, including *LZTFL1*, *SLC6A20*, *FYCO1*, and chemokine receptor genes (*CCR9*, *CXCR6* and *XCR1*). Among these genes, *LZTFL1* is broadly expressed in pulmonary epithelial cells, *FYCO1* is involved in transporting of autophagic vesicles, and *SLC6A20* encodes a sodium transporter interacting with *ACE2*, the receptor that SARS-CoV-2 binds to [18]. Two other robust genetic associations to disease severity point to inflammasome regulator *DPP9* (19p13.3) and high-affinity interferon α/β receptor *IFNAR2* (21q22.11), originally reported by the Genetics of Mortality in Critical Care (GenOMICC) GWAS [20].

One of the first risk loci for COVID-19 severity identified by GWAS was the *OAS* gene cluster (12q24.13 including *OAS1*, *OAS2* and *OAS3*), carrying a Neanderthal-derived haplotype [20]. These *OAS* genes encode proteins involved in viral clearance. A plausible causal variant in *OAS1* (rs10774671) was independently identified by two groups to predict an isoform of *OAS1* using a two-sample Mendelian randomization method [21] and a trans-ancestry fine-mapping approach [22].

On the X chromosome, a non-coding upstream variant of *ACE2* (Xp22.2) was associated with disease susceptibility [23]. Analysis of RNA sequencing data from liver tissue showed that the protective rare rs190509934-C allele downregulates *ACE2* expression and subsequently impacts disease risk [23]. The T allele of rs2285666, an *ACE2* intronic variant, was associated with critical outcomes among male COVID-19 patients [24]. Carrying the rs2285666-T allele was associated with increased risk for critical pneumonia in males with COVID-19 and was linked to impaired type I interferon responses [25].

Many lead variants reported by GWAS of COVID-19 severity are located in non-coding regions and within large haplotype blocks in high linkage-disequilibrium (LD), such as the 3p21.31 locus. CRISPR (clustered regularly interspaced short palindromic repeat) genome editing technology identified *CCR9* and *SLC6A20* as plausible causal genes associated with COVID-19 severity [26], while joint genome-scale CRISPR loss-of-function screens and expression quantitative trait locus analysis pointed to *SLC6A20* and *CXCR6* as target genes [27]. A recent study used a CRISPR technology to link the risk allele of rs11385942 (an intronic variant in *LZTFL1*) with reduced expression of *LZTFL1* in lung epithelial cells [28]. Among these previously reported GWAS findings, associations at *FOXP4* and *LZTFL1/SLC6A20* are present in both the ‘HGI7no’ summary statistics and in our HostSeq results.

## Materials & methods

### Ethics statement

HostSeq was approved by the Research Ethics Board of The Hospital for Sick Children (#1000070720 from 2020-present). Written informed consent was obtained from all participants or parents/guardians/substitute decision makers prior to inclusion in the study.

### HostSeq genotype and phenotype data

We analyzed genetic data from version 9 (v9) of the HostSeq project [1], which was released in March 2023, and included 10,059 genomes from 15 studies across Canada. Recruitment, sequencing and joint-calling details are provided in the HostSeq resource paper [1].

We extracted the following phenotypic variables from the Case Report Forms (CRFs) for our analysis: hospitalization status, sex and age. When an individual’s age was not directly available in CRF, their age was inferred using the dates of birth and sample collection. If the latter was unavailable, June 2020 was used as a proxy endpoint.

For this analysis, we removed 1,585 participants during the quality control and preprocessing steps (described in *Data processing* below and shown in S1 Fig), yielding a sample size of N = 8,474. We note that 1,312 of 1,585 participants were excluded because their phenotype information was either unavailable or insufficient in the harmonized clinical database. Table 1 shows the sample size for each of the contributing studies, as well as sample overlap if multiple studies recruited the same participant.

**Table 1:** Summary of study sizes for N = 8,474 HostSeq samples analyzed. Details about the design of these contributing studies and the institutions and investigators involved are provided in the HostSeq resource paper [1]. Stars * indicate that the study has overlapping samples with another study (due to recruitment in multiple studies). The size of the overlap between CANCOV and Concor-Donor is 11 samples. Between CANCOV and GENCOV: 10 samples. Between GENCOV and Concor-Donor: 2 samples. Between BQC19 and IPCO: 9 samples. Between genMARK and Concor-Donor: 2 samples. Between GenOMICC and GENCOV: 1 sample. Between SCB and genMARK: 3 samples. Removing duplicated samples yields N = 8,474.

Study	Hospitalized	Non- Hospitalized	Total
Alberta Childhood COVID-19 Cohort Study (AB3C)	16	151	167
Convalescent Plasma for COVID-19 Research (Concor-Donor)	27	748	775*
Genetic Markers of Susceptibility to COVID-19 (genMARK)	34	702	736*
Genomic Determinants of COVID-19: Integration of Host and Viral Genomic Data to Understand the COVID-19 Epidemiologic Triangle (GD-COVID)	91	391	482
Host Genetic Factors Underlying Severe COVID-19	9	0	9
Host Genetic Susceptibility to Severe Disease from COVID-19 Infection (AB-HGS)	43	10	53
HostSeq—Canadian COVID-19 Human Host Genome Sequencing Ottawa (LEFT-GEN)	10	34	44
Implementation of Serological and Molecular Tools to Inform COVID-19 Patient Management (GENCOV)	61	874	935*
The IRCM POST-COVID-19 Research Clinic: a multidisciplinary approach to evaluate short and long-term complications of COVID-19 (IPCO)	5	52	57*
Screening Protocol for Detection of Infections and Immunodeficiencies and Characterization of Susceptibility to Infectious Diseases	30	7	37
The Canadian COVID-19 Prospective Cohort Study (CANCOV)	430	577	1007*
The Genetics of Mortality in Critical Care (GenOMICC)	320	7	327*
The Hospital for Sick Children’s COVID-19 Biobank (SCB)	92	158	250*
The Quebec COVID-19 Biobank (BQC19)	2334	1289	3623*
Understanding Immunity to Coronaviruses to Develop New Vaccines and Therapies against 2019-nCoV	3	7	10
**Total with 38 duplicates**	3505	5007	8512

We categorized the 8,474 HostSeq individuals into five major ancestry groups (S2–S4 Figs) using ancestry-inference from *GRAF-pop* [29]: 455 ‘AFR’ African (5.4%), 537 ‘AMR’ Admixed American (6.3%), 519 ‘SAS’ South Asian (6.1%), 654 ‘EAS’ East Asian (7.7%), and 6107 ‘EUR’ European (72.1%); the AFR set combines African-American (1.6%) and African-only (3.7%) groups. In addition, 202 samples remained uncategorized (2.4%).

### Data processing

We implemented a comprehensive quality control (QC) procedure on the multi-ancestry joint-called data of the HostSeq genomes available on the human genome build GRCh38 (S1 Fig). We used *bcftools* (v1.11) [30] to determine our variant exclusion list, *VerifyBAMID2* (v2.0.1) [31] to estimate DNA contamination, average read depth and number of reads, and *PLINK* (v2.0.0) [32] to calculate heterozygosity, test Hardy-Weinberg equilibrium (HWE), perform linkage-disequilibrium (LD) pruning, and conduct principal component analysis (PCA). We used the *R* platform (v3.6.3) [33] to conduct descriptive and statistical analyses as well as to create figures.

We performed multiple rounds of alternating variant and sample QC. First, we applied the GATK hard-filtering protocol (Resources) on the joint-called data to exclude variants with low quality measures (details are in S1 Fig). Then, we used the retained variants and information from the CRF to assess the quality of the samples. Here we checked their genome quality (removing 102 samples with genotyping call rate < 99%, or number of reads < 2E6, or contamination > 3%), sample identity (removing 160 samples because of mismatch between reported and predicted sex, or because they were identified to be duplicates), information in clinical database (removing 695 samples because their phenotypic information was not yet harmonized, or because their consent for research was withdrawn), phenotype availability (removing 617 samples due to missing age, sex or hospitalization status), and heterozygosity (removing 11 outliers). After the sample QC we used the retained samples to assess the quality of the variants and removed variants with genotyping call rate < 98%. We then performed principal component analysis on a total of 8,474 samples passing the above QC checks, after LD pruning the variants (details are in S1 Fig). We did not find any extreme sample outliers to remove based on PCA. Finally, we checked for variants with deviations from Hardy-Weinberg Equilibrium (HWE) using the non-hospitalized (control) sample of European ancestry, and removed deviating variants with P < 1E-50 from all samples used in the analyses. S1 Fig provides details for the variant and sample QC criteria. In our summaries of GWAS results (described below), we further excluded variants in difficult-to-sequence regions as annotated by the Genome-In-A-Bottle consortium (GIAB v3.3) [34].

Our comprehensive QC resulted in a final set of ~153M variants and N = 8,474 individuals. S5 Fig shows the quality of the retained samples with regards to missingness, contamination and coverage. The samples include multiple ancestries, as well as some related individuals.

### Genetic analysis variables

Our phenotype of interest is COVID-19 severity as defined by hospitalization status (yes/no), where both cases and controls were SARS-CoV-2 positive. Covariates of interest include age, sex, age x sex, age^2^, age^2^ x sex, and seven genetic PCs; this list of covariates is often used in GWAS [35]. Due to the extensive QC conducted earlier, there were no missing phenotypes or covariates for any of our N = 8,474 samples.

We identified seven important genetic PCs from the scree plot of the final round of PCA (scree plot, pairwise PCA plots, and distribution of PCs are shown in S6–S8 Figs). We created a standardized age variable, defined as (age-50)/10 [36]. We included age^2^ as a covariate because the incidence of hospitalization for COVID-19 may increase non-linearly with age. Sex is also an important risk factor for hospitalization, so in addition to its main effect, we also included age x sex and age^2^ x sex interaction terms as covariates.

### Genome-wide association analyses: Single-variant, G x Sex and gene-based

We used *regenie* (v3.2.9;37) for single-variant association study, interaction testing and gene-based analysis (see S9 Fig for details). Single-variant GWAS is our primary analysis, but accounting for G x Sex (genotype by sex) interaction is also of interest, as the risk for severe COVID-19 differs between males and females [11]. Furthermore, we performed a rare-variant gene-based test because individual coding variants are typically rare, and power to detect association with each single rare variant is low. Such a joint analysis of multiple coding variants in a gene is a commonly employed approach to improve power.

Analyses were performed on bi-allelic variants across chromosomes 1–22 and X. The X chromosome was analyzed separately for the pseudo-autosomal regions (PAR) and non-pseudo-autosomal regions (NPR). In total, 147M autosomal, 0.14M PAR and 6M NPR variants were analyzed. All autosomal and PAR variants, in both males and females, were coded additively as 0, 1 and 2. NPR variants in females were also coded as 0, 1 and 2, but in males, they were coded as 0 and 2, which assumes X-inactivation, as specified by *regenie*. We used the recommended block-size of 1,000 and default parameter values in all *regenie* steps.

The *regenie* implementation involves two steps. Step one uses a subset of variants that “captures a good fraction of the phenotype variance attributable to genetic effects” [37] and forms phenotype predictions [37], and step two performs the association analysis conditional on the predictions from step one. For step one, we prepared the required subset by restricting variants to Illumina’s Global Screening Array (GSA v3 b151 GRCh38; Resources) with minor allele frequency (MAF) > 10% and minor allele count (MAC) > 100. Using the predictions from step one, we executed step two to obtain our primary GWAS results, as well as G x Sex interaction and gene-based testing results. In the second step of regenie, we opted for the Firth-approximation option for more accurate association p-value calculation. For the G x Sex interaction analysis, we report results from jointly testing for G main and G x Sex interaction effects. This two degrees-of-freedom (2 d.f.) joint test is better powered to detect variants with sex-specific genetic effects, and in the absence of effect heterogeneity, it is comparable to the standard GWAS approach of testing for main effect only [38].

For the gene-based analysis inclusive of rare variants, we first annotated coding regions outside difficult-to-sequence regions using *Ensembl* (v110.1; 39) and selected variants with high/moderate impact. We then performed the SKAT-O test [13] as implemented in *regenie* with the default weighting factors of a1 = 1 and a2 = 25. Coding variants in 17,886 genes were categorized into two regenie masks: (i) high impact and (ii) high/moderate impact. We performed the test twice with maximum alternate allele frequency (AAF) set to 0.01 or 0.05, as estimated from HostSeq.

### Genome-wide association analyses: Single-variant meta-analysis

Although our primary single-variant HostSeq-wide analysis via regenie accounted for population stratification, as a complementary alternative approach, we additionally conducted a meta-analysis with stratification across ancestry groups. To this end, we first used *SAIGE* [9] to obtain single-variant summary statistics for each ancestry group. We then meta-analyzed these ancestry-specific GWASes using *MR-MEGA* [10].

More specifically, within each of the five ancestry groups (N: AFR = 455, AMR = 537, SAS = 519, EAS = 654, and EUR = 6,107), we conducted GWAS using the *SAIGE* mixed model. This involved incorporating a kinship matrix as a random effect and covariates as fixed effects. *SAIGE* uses Firth’s Bias-Reduced Logistic Regression to estimate effect sizes and the saddlepoint approximation to calibrate unbalanced case-control ratios, which is essential for some ancestry groups (S1 Table). Similar to the primary mega-analysis, the ancestry-stratified GWAS here was restricted to bi-allelic variants across the whole genome including the X chromosome, and accounting for the same set of covariates. But considering the smaller ancestry-specific sample size and *SAIGE* recommendations, we further removed variants with MAF < 1%, MAC < 20, and genotyping call rate < 99%. To aggregate the above ancestry-specific GWAS summary statistics (total N = 8,272), *MR-MEGA* considers the potential heterogeneity in effect sizes across ancestry groups. To be conservative, *MR-MEGA* first applies a genomic control correction to each ancestry-specific GWAS to account for residual population structure before meta-analysis. It then includes the first two PCs, derived from a matrix of allelic frequency similarities between GWASes, as covariates. We note that, unlike the regenie analysis (N = 8,474), we did not include the 202 individuals without clear ancestry categorization from the meta-analysis, because *MR-MEGA* may not be effective when analyzing admixed individuals [10].

### Comparison to HGI and functional analysis

Using our HostSeq association results for COVID-19 severity we aimed to replicate HGI findings for the B1 contrast. The available HGI GWAS summary statistics are from a meta-analysis of several studies including two studies from HostSeq (BQC19 and CGEN). Therefore, we sought to subtract out the effect due to sample overlap using the *R* package *MetaSubtract* (v1.60;40). To achieve this, we used the available ‘leave-one-out BQC19’ HGI GWAS results (which also does not include 23andMe) and further removed the effect of CGEN using MetaSubtract. We refer to this HGI v7 non-overlapping version as HGI7no (cases = 15,591, controls = 70,608). We used HGI7no findings for our replication study. We identified three genome-wide significant loci in HGI7no and examined their colocalization in HostSeq through *myLocusZoom* (v0.14.0) [41,42] and *LocusFocus* (v1.4.9 alpha) [43]. The *LocusFocus* colocalization tool considered a local area around each lead variant spanning 0.1Mb on either side (including 300–600 variants for each lead variant). Additionally, we performed functional analysis using *MAGMA* (v1.08) [44] as implemented in the *FUMA GWAS* (Functional Mapping and Annotation of Genome-Wide Association Studies v1.6.1; 12) software package.

### Polygenic risk score

We used *PRSice-2* (v2.3.5) [45] to calculate polygenic risk scores (PRS) using HGI7no as our base data (see S9 Fig for details). We calculated standardized PRS (scaling so that mean = 0 and SD = 1) using the three variants in HGI7no that passed the genome-wide significance level of p-value < 5E-8 [46] after LD-clumping (window-size = 750kb, r^2^ = 0.1). To determine the extent of polygenicity in our study, we also calculated PRS using HGI7no at additional p-value thresholds of 1E-5, 1E-4, 1E-3, 1E-2, 5E-2, 1E-1, 5E-1, and 1; the X chromosome is not included in the *PRSice* computation.

*PRSice* is a clumping and thresholding method which works by selecting a single variant with the highest p-value from LD blocks constructed with the target population. This process is prone to discarding potentially relevant information (especially when considering a large number of SNPs) and imposes constraints on the genetic architectures that can be modeled [45]. To complement this clumping and thresholding approach employed by PRSice, we also applied an alternative method, *PRS-CS* (v1.1.0) [47]. In this approach, the weights assigned to SNPs in the PRS are updated based on their association strength in the GWAS using a Bayesian framework. The advantages of using this approach are: (i) flexibility in accommodating diverse genetic architectures, especially considering the unknown architecture of COVID-19 severity, and (ii) integration of information from an external reference panel for LD patterns, as previous studies [48] have demonstrated its efficacy in enhancing predictive performance.

Specifically, we used *PRS-CS* to adjust the effect sizes of autosomal SNPs present in both HGI7no and HostSeq based on the LD reference panel [47] pre-computed by *PRS-CS* from the European super-population of the 1000 Genomes Project phase 3 (Resources). We allowed for the global shrinkage prior (the ϕ parameter) to be estimated from the data using a fully Bayesian approach. Finally, we used these modified effect sizes to calculate PRS using *PLINK* (v1.9) [49].

### Heritability

We used *LDSC* (v1.0.1) [50] to estimate SNP-based heritability in HostSeq and HGI. Summary statistics were quality-controlled using the mungeStats pipeline recommended by *LDSC* (using the LD scores from the 1000 Genomes Project phase 3); the X chromosome is not included in the *LDSC* computation.

## Results

Table 2 shows the basic demographics of the N = 8,474 HostSeq v9 participants analyzed. As expected, age was significantly associated with COVID-19 hospitalization status, with older individuals having a higher risk (Welch two-sample t-test: P < 2.2E-16; T = -43.93; S10 Fig). Sex at birth was also associated with being hospitalized with females having a lower risk (Fisher’s exact test: P < 2.2E-16; OR = 0.41; 95% CI = [0.37, 0.45]). S1 and S2 Tables provide counts by sex and hospitalization status, stratified by ancestries and studies, respectively. S11 Fig compares the allele frequency distribution between HostSeq and gnomAD (v3.1.2) [51], where HostSeq samples are the 100% European ancestry (as predicted by *GRAF-pop*) subset and gnomAD samples are the non-Finnish European subset.

**Table 2:** Summary statistics for the N = 8,474 samples in the examined HostSeq study cohort. All samples were COVID-19 positive. While females were slightly more represented in the recruited population, more than half of the hospitalized participants were males.

		Hospitalized	Non-Hospitalized	Total
**Sample size**		3,499	4,975	8,474
**Age (years)**	**Mean (SD)**	59.31 (20.65)	40.84 (16.52)	48.46 (20.47)
**Sex**	**Male**	1,954 (55.8%)	1,692 (34.0%)	3,646 (43.0%)
	**Female**	1,545 (44.2%)	3,283 (66.0%)	4,828 (57.0%)

### Genome-wide association analyses: Single-variant

**Fig 1:**
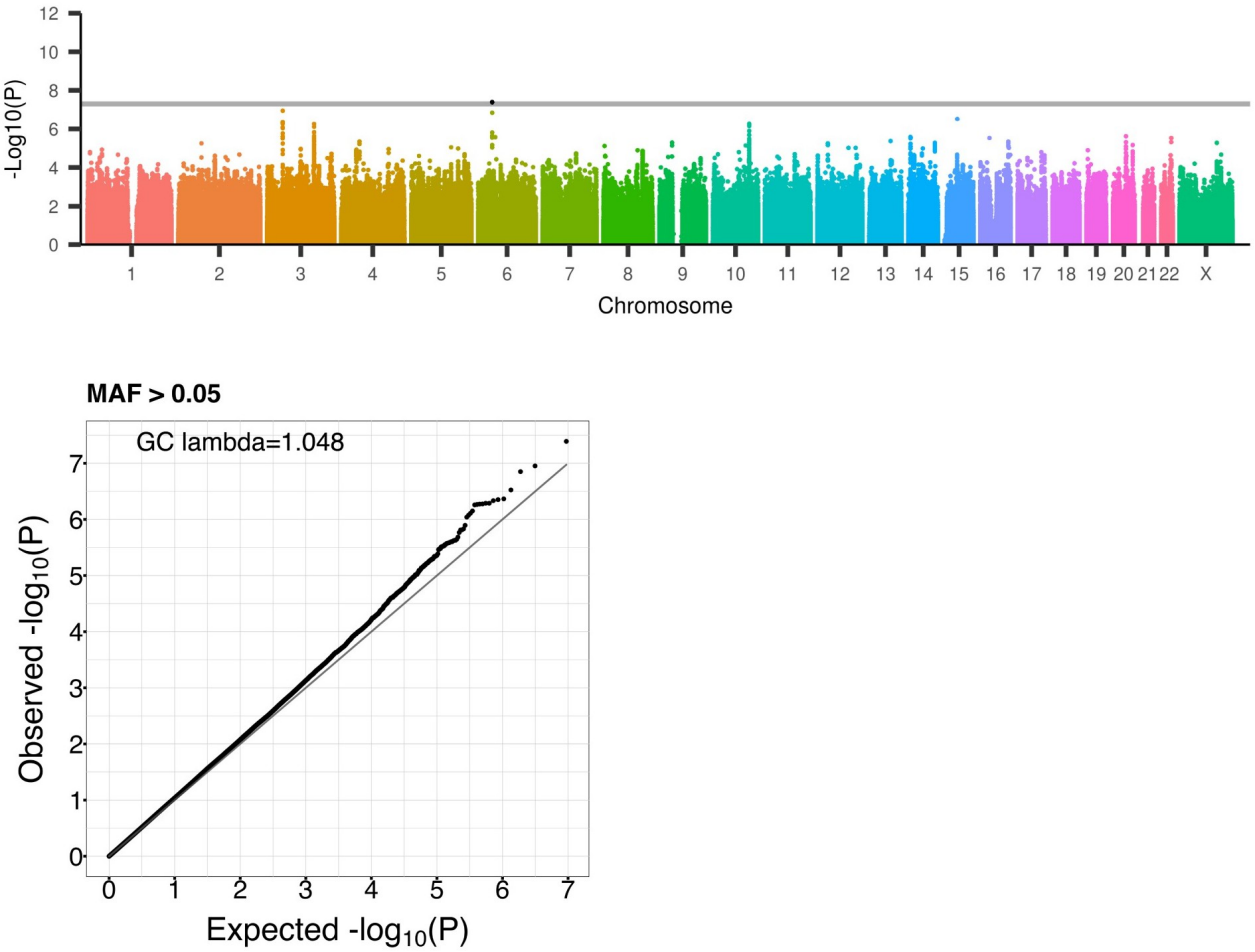
Genome-wide association study of hospitalization status in 8,474 HostSeq samples with COVID-19 from the March 2023 release (V9). In the Manhattan plot, Y-axis indicates -Log10 p-values of regenie analysis for variants with MAF > 5%, X-axis indicates chromosomes. Variants falling in the GIAB difficult-to-sequence regions have been excluded. Grey horizontal line indicates genome-wide significance level of P < 5E-8. Chromosome 6 and chromosome 3 loci have been previously identified in HGI. In the corresponding QQ-plot, the X and Y axes indicate expected and observed -Log10 p-values, respectively (genomic control λ = 1.048).

Fig 1 shows the primary HostSeq GWAS results from regenie for variants that are not in difficult-to-sequence regions and with MAF > 5% (genomic control inflation statistic, λ = 1.048). MAF-stratified QQ-plots and p-value histograms are provided in S12 Fig and show that the study has a well-controlled type I error rate by focusing on MAF > 5%. S13 Fig presents the results prior to the removal of difficult-to-sequence regions, showing the importance of excluding variants in difficult-to-sequence regions as part of QC since sporadic signals appear in some of these regions.

Here we report the top five HostSeq loci, including one genome-wide significant hit on chromosome 6 (Table 3). Of the five variants, rs4714474 (chr6:41535823 on 6p21.1) and rs35731912 (chr3:45848457 on 3p21.31), are respectively in LD (Resources) with lead variants reported by HGI at the *FOXP4* (rs12660421) and *LZTFL1* loci (rs17713054). S14 Fig shows the surrounding regions in *myLocusZoom* for the other three variants. These are rs78173596 (chr15:54131608 on 15q21.3), an intronic variant of *UNC13C*, rs17122332 (chr10:107238146 on 10q25.1) an intergenic variant upstream of *SORCS1*, and rs1199346 (chr3:138353967 on 3q22.3) an intronic variant of *MRAS*. eQTLGen Phase I [52] also identifies the chromosome 3 hits to be significant cis-eQTLs (rs35731912 of *FLT1P1*, *CCR3*, *CXCR6*, *CCR1*, *SACM1L*, *CCR5*, *CCR9*, *CCR2* and *RP11-24F11*.*2*; rs1199346 of *MRAS*, *CEP20* and *FAIM*).

**Table 3:** Association details of lead variants from HostSeq. Top loci in HostSeq after applying a MAF > 5% filter and removing variants in the GIAB difficult-to-sequence regions. Chromosome 6 hit passes the genome-wide significance threshold of P < 5E-8, and is in LD with a HGI7no lead variant (rs2496646: D’ = 0.87; r^2^ = 0.42). Chromosome 3 hit is also in LD with a HGI7no lead variant (rs17763742: D’ = 0.95; r^2^ = 0.82). Nearest-gene annotation is from myLocusZoom. Multi-ancestry meta-analysis p-values of HostSeq are added in paranthesis after primary HostSeq results (MR-MEGA); ‘m’ indicates number of ancestries MR-MEGA used for the result.

Marker	rs4714474	rs35731912	rs78173596	rs17122332	rs1199346
**Chromosome**	6	3	15	10	3
**Position**	41,535,823	45,848,457	54,131,608	107,238,146	138,353,967
**Nearest-Gene**	FOXP4-AS1	LZTFL1	UNC13C	SORCS1	MRAS
**Effect Allele**	A	T	C	G	A
**Reference Allele**	G	C	T	A	G
HostSeq					
**Effect Allele Freq.**	0.07	0.10	0.10	0.15	0.79
**Beta**	0.47	0.37	0.36	-0.29	-0.26
**SE**	0.09	0.07	0.07	0.06	0.05
**P-value**	4.1E-08 (8.3E-7, m = 4)	1.1E-07 (1.1E-7, m = 5)	3.0E-07 (1.4E-6, m = 3)	5.4E-07 (1.5E-4, m = 5)	5.5E-07 (5.1E-5, m = 5)
HGI7no					
**Effect Allele Freq.**	0.07	0.16	0.12	0.12	0.78
**Beta**	0.30	0.36	0.05	0.02	0.01
**SE**	0.05	0.03	0.03	0.03	0.03
**P-value**	3.5E-11	1.3E-29	1.4E-1	5.0E-1	7.4E-1

Additionally, we performed functional analysis using *MAGMA* as implemented in *FUMA GWAS* [12]. The gene-based test as computed by *MAGMA* found *MRAS* with P = 3.52E-7 to be genome-wide significant at ɑ = 0.05/18,329 = 2.73E-6 (S15 Fig). Furthermore, the *MAGMA* gene-set analysis found a curated gene set ‘HASEGAWA_TUMORIGENESIS_BY_RET_C634R’ comprising 7 genes to be significant with P = 4.02E-4 after Bonferroni-correction (S3 Table provides results for each of the 7 genes).

The multi-ancestry meta-analysis did not reveal any new loci (genomic control inflation statistic, λ = 0.991; S16 Fig). *MR-MEGA* meta-analysis of the five categorized ancestries included an ancestry-specific genomic control correction on summary statistics from *SAIGE* (λ_gc_: EAS = 1.05; AMR = 1.06; EUR = 1.00; ​​AFR = 1.05; SAS = 1.04). Result for the *LZTFL1* locus (rs35731912: P = 1.13E-7) is similar to the primary GWAS but results for the *SORCS1* (rs17122332: P = 1.54E-4) and *MRAS* loci (rs1199346: P = 5.09E-5) are less significant (Table 3). We note that *MR-MEGA* meta-analysis only reports results for variants that have a *SAIGE* result in each of the five ancestries.

**Fig 2:**
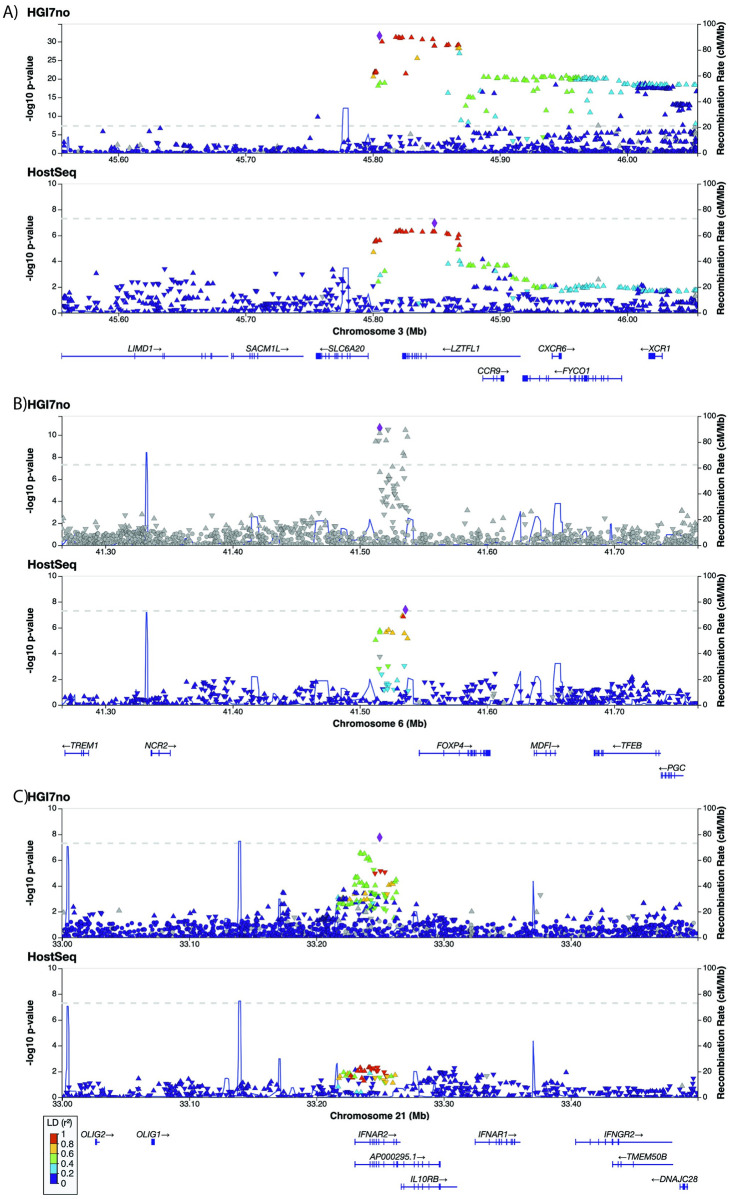
Region plots for the top three loci from HGI7no compared with HostSeq. Querying the three regions: a) chr3:45805277, b) chr6:41515629, c) chr21:33249643 in HGI7no (top row in each pane) with HostSeq (bottom row in each pane) shows similar patterns for two out of three loci (chr3:45805277, chr6:41515629). Plots were generated using myLocusZoom.

**Fig 3:**
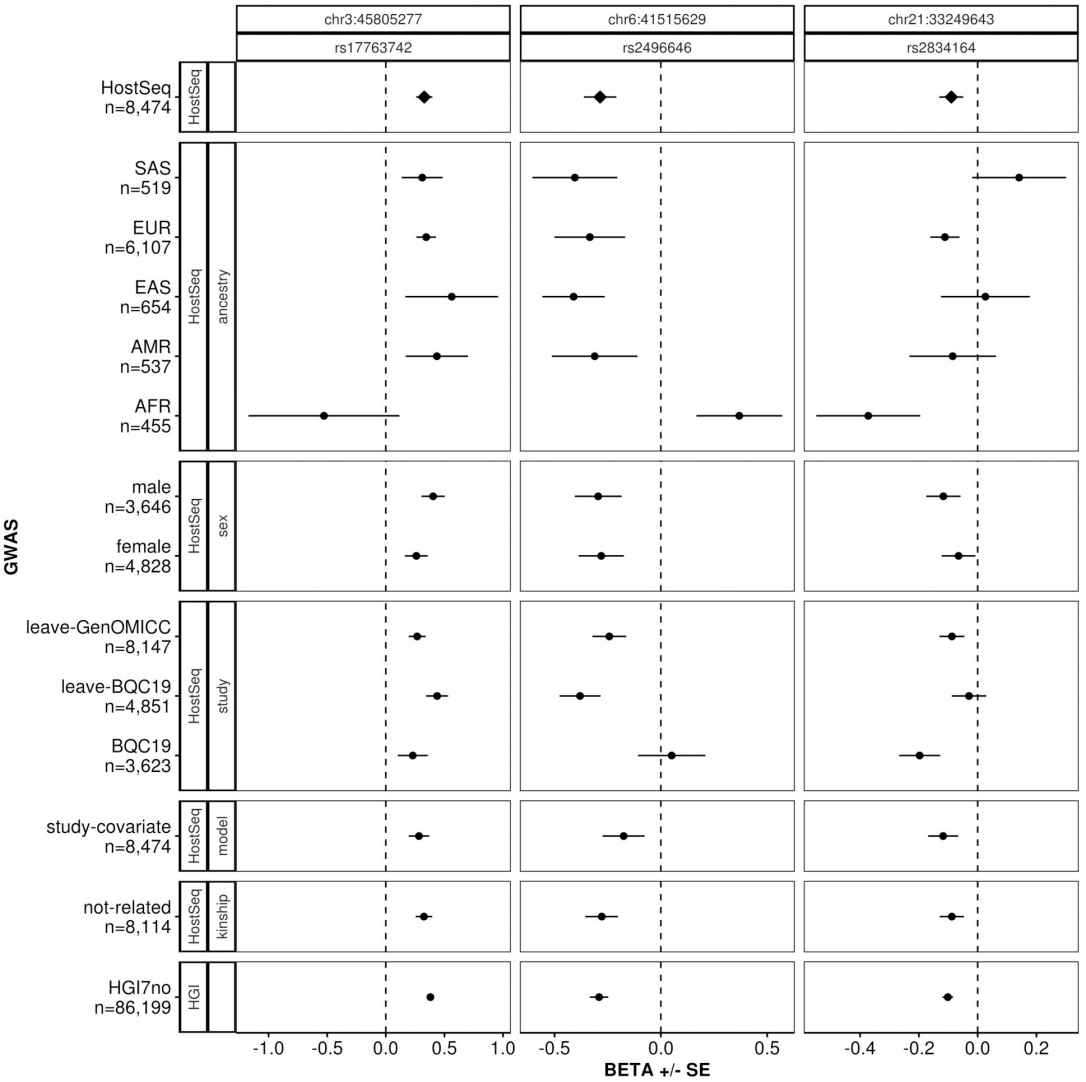
Within-HostSeq comparison of the three lead variants from HGI7no. Examination of the three lead variants from HGI7no, depicting beta and SE for all N = 8,474 HostSeq samples and various stratifications of the HostSeq samples. The top panel shows results for all HostSeq samples passing QC. The last panel shows results from HGI7no. The panels in between show results for various stratifications of HostSeq including ancestry, sex, study, model and kinship. In the ‘model’ panel, ‘study-covariate’ indicates that HostSeq study centre was added as a categorical covariate to the main model (top panel). In the ‘kinship’ panel, ‘not-related’ indicates the subset that excludes samples within 2 degrees of relatedness as determined by KING. At the chromosome 3 locus all subsets, except for AFR, are consistent in sign of beta (i.e. effect direction). At the chromosome 6 locus, in addition to AFR, BQC19 differs from all other HostSeq subsets in sign of beta. The chromosome 21 locus is the most variable within HostSeq. Note that the X-axis scale varies among the three variants.

We then compared the three HGI7no lead variants that were genome-wide significant to the primary HostSeq GWAS results using *myLocusZoom*. Fig 2 shows that out of the three loci (chr3:45805277, chr6:41515629 and chr21:33249643 on 3p21.31, 6p21.1 and 21q22.11, respectively), the patterns at two loci (on chromosomes 3 and 6) colocalize between the two studies. A formal analysis using *LocusFocus* revealed that the colocalization is statistically significant with p-values of 6.46E-7, 2.09E-6 and 0.007 for chr3:45805277, chr6:41515629 and chr21:33249643, respectively. For each locus, the colocalization is further supported by the consistent variant effect sizes and directions between HGI and HostSeq (Table 3 and Fig 3). A power calculation (Resources) shows that in HostSeq, the power to replicate the three loci at ɑ = 0.05/3 = 0.0167 are 100%, 100% and 84.2% for chr3:45805277, chr6:41515629, and chr21:33249643, respectively. S4 Table provides a comparison between HGI7no and HostSeq for 47 of the 51 hits reported by HGI (Table 2 of [8]) that were present in HGI7no. This Table includes rs190509934 on *ACE2* which is reported in HGI; this variant is not significant in HostSeq but its effect size is directionally consistent with HGI7no.

To examine within-HostSeq consistency for these three variants, we performed additional association analyses using various subsets: (i) stratified by ancestry, (ii) stratified by sex, (iii) stratified by study and leave-one-out study, (iv) unrelated samples up to 2 degrees away. For the leave-one-out study subsets we chose the BQC19 and GenOMICC studies to be sequentially excluded, because BQC19 is the largest study within HostSeq and accounts for more than half of the cases in HostSeq [53], and GenOMICC is the most restricted study in terms of recruitment and predominantly consists of hospitalized cases. Furthermore, we tested these three variants in a different model, by adding study as a categorical covariate. Fig 3 shows that the effect directions for all three variants are consistent with a few notable exceptions: African-ancestry subset stands out for all three variants; the BQC19-study subset is inconsistent with the rest of the cohort at the chromosome 6 locus; and the chromosome 21 locus has the smallest effect size and is the most variable across ancestry-subsets. The ancestry composition of BQC19 is diverse but has a higher proportion of European and African samples than HostSeq overall, which may be affecting results: 231 African (6.4%), 240 Admixed American (6.6%), 118 South Asian (3.3%), 166 East Asian (4.6%), 2813 European (77.6%), and 55 uncategorized (1.5%). Note that the African-ancestry subset is a heterogeneous group consisting of admixed African-American groups as well as diverse African groups. S5 and S6 Tables provide allele frequencies of the top three HGI7no variants, stratified by ancestries and studies, respectively.

### Genome-wide association analyses: G x Sex and gene-based

The G x Sex interaction analysis did not yield any genome-wide significant results (genomic control inflation statistic, λ = 1.194; S17 Fig). We note that the interaction analysis conducted was based on the 2 d.f. joint testing of G main and G x Sex interaction effects, which is more robust to model misspecification than the interaction analysis alone and, in the absence of interaction effects, provides comparable results with the main effect GWAS [38]. Indeed, the Manhattan plot in this analysis (S17 Fig) is similar to the Manhattan plot of the primary GWAS (Fig 1). Specifically, two of the top hits in this analysis (P_rs4714474_ = 2.08E-7, P_rs35731912_ = 2.42E-7) are the same SNPs as in the primary GWAS at the *FOXP4* and *LZTFL1/SLC6A20* loci. Additionally, this analysis identified a locus with suggestive sex-specific effects, although the association evidence at the lead SNP rs79973703 (chr7:107127037 on 7q22.3; P = 9.33E-8) did not reach genome-wide significance. This variant is an intronic variant of *PRKAR2B*, and a significant cis-eQTL of *COG5*, *AC002467*.*7*, *HBP1* and *PIK3CG* [52]. S18 Fig compares this locus with sex-stratified results through region plots on *myLocusZoom* and shows that the effect is driven by an association in the male-subset of HostSeq which has β = -0.50 and P = 3.21E-7 (S7 Table).

The genome-wide gene-based SKAT-O investigation produced results for two *regenie* masks: (i) 3,351 genes in high impact, (ii) 17,342 genes in high/moderate impact (S19 Fig). A protein-coding gene on chromosome 14 (14q23.2), *WDR89*, passed genome-wide significance (P = 1.89E-10) in the high/moderate impact tests with both no alternate allele frequency (AAF) filter and a maximum AAF 5% filter. *WDR89* encompassed 23 missense variants of moderate impact. S8 Table shows the results of 7 of these variants which had a non-NA (not available) p-value in the unfiltered (without the MAF restrictions and difficult-to-sequence screening) primary GWAS. Three of these 7 variants, which are within an 8 bp region and in LD with each other, are significant in the unfiltered primary GWAS (chr14:63599677, chr14:63599680, chr14:63599684) with MAF around 2% and the lowest p-value being 9.56E-11. These variants are in the third and final exon of *WDR89*. Removing all three variants (chr14:63599677–63599684) resulted in a non-significant SKAT-O result for *WDR89* (P = 0.855), and adding chr14:63599684 back made it significant again (P = 1.93E-10), illustrating the effect of this sub-region. Notably, in gnomAD (v4.0; 51) all three of these variants have similar MAF at 2–3% but failed gnomAD quality control measures (specifically AS_VQSR, which is an allele-specific quality control protocol) in both exome and genome sequence data, raising concerns about their quality. The HostSeq quality measures for these three variants and all of the other variants discussed in Tables 3 and 4 are provided in S9 Table. No other gene-based analysis produced genome-wide significant results, including *ACE2* (SKAT-O result for 44 moderate impact variants: P = 0.25).

**Table 4:** Association details of the three lead variants from the HGI7no GWAS (N = 86,199) compared with the HostSeq study (N = 8,474). Effect direction and magnitudes are consistent between HGI7no and HostSeq at the three loci (chr3:45805277, chr6:41515629, chr21:33249643). Nearest-gene annotation is from myLocusZoom. Multi-ancestry meta-analysis p-values of HostSeq (MR-MEGA) are added in paranthesis after primary HostSeq results; ‘m’ indicates number of ancestries MR-MEGA used for that result.

Marker	rs17763742		rs2496646		rs2834164	
**Chromosome**	3		6		21	
**Position**	45,805,277		41,515,629		33,249,643	
**Nearest-Gene**	SLC6A20		FOXP4-AS1		IFNAR2	
**Effect Allele**	G		C		C	
**Reference Allele**	A		T		A	
**Study**	HGI7no	HostSeq	HGI7no	HostSeq	HGI7no	HostSeq
**Effect Allele Freq.**	0.16	0.10	0.85	0.91	0.43	0.48
**Beta**	0.38	0.33	-0.29	-0.29	-0.10	-0.09
**SE**	0.03	0.07	0.04	0.08	0.02	0.04
**P-value**	2.4E-32	2.5E-6 (2.4E-7, m = 3)	2.2E-11	1.8E-4 (6.1E-5, m = 5)	1.7E-8	2.9E-2 (2.2E-2, m = 5)

### Polygenic risk scores

We constructed a PRS using the three variants that passed the stringent threshold of genome-wide significance (P < 5E-8) and LD-clumping in HGI7no. These are the same three lead variants of HGI7no that are described in Table 4. As expected, our PRS was significantly associated with the hospitalization status (P = 5.25E-13), and explains 1.01% proportion of variance after accounting for all the covariates as shown in Table 5. S10 Table shows the results of the PRS with PCs excluded from the list of covariates. Both the model with PCs and without PCs maintain the signal, providing evidence that population structure does not confound our analysis.

**Table 5:** Association of PRS with hospitalization status accounting for covariates and PC effects. PRS was constructed using the three variants that passed the genome-wide significant P < 5E-8 threshold. The PRS association is significant (P = 5.25E-13) after controlling for genetic PCs and covariates (where sex is coded as males = 1 and females = 2, and age is standardized as (age-50)/10). Proportion of variance explained by PRS is 1.01% (calculated as 1-(1-R^2^_full_)/(1-R^2^_null_), where R^2^_full_ and R^2^_null_ represent R^2^ of models with and without the PRS, respectively).

Term	Beta	SE	T-statistic	P-value
**Intercept**	0.457	0.113	4.06	4.81E-05
**PRS**	0.215	0.030	7.22	5.25E-13
**Sex**	-0.840	0.070	-12.00	3.43E-33
**Age**	0.629	0.058	10.93	8.75E-28
**Age** ^**2**^	0.074	0.021	3.55	3.89E-04
**Age x Sex**	0.034	0.036	0.96	3.37E-01
**Age**^**2**^ **x Sex**	0.044	0.013	3.26	1.11E-03
**PC1**	52.668	2.693	19.56	3.66E-85
**PC2**	19.465	2.497	7.79	6.49E-15
**PC3**	21.261	2.473	8.60	8.22E-18
**PC4**	13.487	2.410	5.60	2.18E-08
**PC5**	-22.048	2.569	-8.58	9.29E-18
**PC6**	35.459	2.585	13.72	7.83E-43
**PC7**	7.459	2.639	2.83	4.71E-03

PRS calculated at additional p-value thresholds yielded significant R^2^ at the ɑ = 0.05 level for P < 1E-5 (53 SNPs were included in the PRS at this threshold), and was significant under Bonferroni correction. However, as more SNPs were included in the PRS, the significance and R^2^ lowered S11 Table.

In the alternative *PRS-CS* approach a total of 1,033,441 SNPs were analyzed, but there was no improvement in association result with hospitalization status (P = 4.63E-5) over the *PRSice* analysis of top three loci (S11 Table).

### Heritability

SNP-based heritability estimates were calculated using LDSC to determine the extent to which genetics impact COVID-19 severity in the HostSeq dataset. The SNP-heritability was estimated to be h^2^ = 0.0159 (se = 0.0484) in the HostSeq dataset, similar to the HGI counterpart (h^2^ = 0.016, se = 0.0045). Version numbers, links, and references for all software used in this study are provided in Table 6.

**Table 6:** Software resources. Version numbers, links, and references for the software packages used in this study.

Process	Software	Version	URL	Reference
GWAS	regenie	3.2.9	https://rgcgithub.github.io/regenie/	[37]
Variant annotation	ensembl-vep	110.1	https://useast.ensembl.org/info/docs/tools/vep/index.html	[39]
Querying loci of interest	myLocusZoom	0.14.0	https://my.locuszoom.org/	[41]
Colocalization	LocusFocus	1.5.0 alpha	https://locusfocus.research.sickkids.ca/	[43]
Functional analysis	FUMA GWAS	1.6.1	https://fuma.ctglab.nl	[12]
PRS	PRSice	2.3.5	https://choishingwan.github.io/PRSice/	[45]
Power calculation	Genetic Association Study (GAS) Power Calculator	2017	https://csg.sph.umich.edu/abecasis/gas_power_calculator/	[59]
Remove a GWAS from meta-analysis	MetaSubtract	1.60	https://cran.r-project.org/web/packages/MetaSubtract/index.html	[40]
GWAS	SAIGE	1.3.0	https://saigegit.github.io/SAIGE-doc/	[9]
GWAS Meta-analysis	MR-MEGA	0.2	https://genomics.ut.ee/en	[10]
PRS	PRS-CS	1.1.0	https://github.com/getian107/PRScs	[47]
Heritability estimation	LDSC	1.0.1	https://github.com/bulik/ldsc	[60]

## Discussion

Genetic variants found to be associated with COVID-19 severity or susceptibility may implicate genes in biological pathways relevant to the SARS-CoV-2 virus. Genetic associations for other infectious diseases have often led to drug targets and drug discovery [3,54]. Therefore, host genetics can inform therapeutics and treatment by suggesting targets for drug development.

In this work, we present a GWAS of COVID-19 severity in HostSeq, a Canadian WGS cohort. Our HostSeq GWAS replicated two main loci from the HGI meta-analysis. However, there are some limitations to our analysis. First, the HostSeq participating studies recruited individuals in different ways, and have variable proportions of hospitalized cases (Table 1). Thus, unweighted logistic regression (as implemented in *regenie* for example) does not produce unbiased estimates (and standard errors) of regression coefficients. Although, studies in other areas [55] suggest the bias may not be large for the estimation of genetic effects when genotypes are unrelated to the probability of recruitment, this assumption is not straightforward to verify. Second, the participating studies are heterogeneous in the relative proportions of cases and controls (see Table 1). The effect of combining them into a single study is not fully understood, and was discussed previously in our resource paper [1]. In this paper we examined the issue of study heterogeneity through various sensitivity analyses (Fig 3) which suggest that our study is not confounded. However, further exploration may improve study power. The third challenge is the overlap of samples between HostSeq and HGI. Since HostSeq consists of several independent studies, two studies had independently submitted their B1 GWAS results to HGI (BQC19 and CGEN) and were included in the HGI v7 meta-analyses. Therefore, the publicly-available HGI meta-analysis results are not completely independent of HostSeq. We were able to utilize HGI’s summary statistics from their leave-one-out analyses to exclude BQC19 and CGEN and obtain the HGI7no summary statistics independent of our HostSeq study. However, a limitation of using the leave-one-out HGI meta-analyses is that the publicly available versions additionally excluded one of their largest studies (23andMe), reducing the HGI7no cohort size to 86,199 and dampening the association results. The omission of 23andMe results from the HGI meta-analysis results could have also limited the development of PRS using PRS-CS, which becomes increasingly powerful as the number of participants in the base GWAS increases beyond 100,000 [47]. Finally, there is a limitation for our ancestry- and sex-specific analyses due to unavailability of parallel results from the HGI B1 contrast. The lack of ancestry-specific GWAS results also precluded the use of PRS software specifically designed for cross-ancestry analyses, such as *PRS-CSx* [56]. Since we could not create ancestry-specific PRS, we provided PRS association results stratified by ancestry post-computation.

Our analysis aimed to include all individuals available including related individuals and individuals from diverse ancestries. To ensure the validity of our analysis, we performed rigorous quality control where we checked samples for their heterogeneity as well as principal component scores so that we would be able to include all the samples that passed these filters. Due to our comprehensive QC and use of *regenie* (which employs a genetic relatedness matrix), we did not exclude samples due to their ancestry or relatedness, or apply genomic inflation adjustments in our primary analysis (as confirmed by QQ-plots and λ_gc_ genomic control estimate). Nevertheless, we performed additional analyses to show the effect of ancestry-stratification and kinship-restriction on the three variants that we sought to replicate from HGI7no. These additional analyses show the robustness of our primary results, and yield further evidence of replication.

Our GWAS analysis also included the often overlooked X chromosome [57] and considered G x Sex interaction. Although neither analyses led to genome-wide significant results, there was one suggestive finding from the G x Sex interaction analysis. We found a locus on chromosome 7 which has an effect driven by males. Inclusion of the X chromosome allowed us to investigate *ACE2*, but unsurprisingly as the variant reported by HGI is a rare variant and HGI suggested that the association is with infection susceptibility, we did not find any association with disease severity in our study of N = 8,474. For future studies of larger cohorts, analyzing the X chromosome and testing G x Sex interactions are worthwhile considerations.

## Conclusion

In this work, we investigated 10,059 participants from the multi-ancestry and Canada-wide HostSeq. Of these, N = 8,474 participants passing quality control were analyzed. Our GWAS replicated two (*LZTFL1/SLC6A20* and *FOXP4*) out of three loci that were reported in Version 7 of HGI for the B1 contrast. The third locus (*IFNAR2*) has a relatively smaller effect size and is directionally inconsistent among the HostSeq ancestries, which contributes to its diminished overall effect. The standard errors of effect estimates for all three variants are larger in the African-ancestry subset relative to the other ancestry groups. This is likely due in part to the smaller sample size (N = 455), lower MAC, and heterogeneity within this group. This may also be due to differences in risk factors across studies and ancestry groups. More importantly, it may be caused by the within-group diversity of the African-ancestry subset, which includes both recently admixed populations (African-American) and African-only groups (see S3 Fig).

Our GWAS led to a genome-wide significant locus in LD with the known *FOXP4* locus. Gene-based analyses identified two significant genes, *MRAS* (from *FUMA GWAS*), and *WDR89* (from SKAT-O). Examination of genotype-by-sex effects for host genetics of COVID-19 severity did not lead to genome-wide significant novel loci, but we did find a locus with sex-specific effects.

We also examined heritability and constructed a polygenic risk score (PRS) using summary statistics. Heritability estimates were found to be almost identical between the HostSeq and the HGI7no dataset (h^2^ = 0.0159, h^2^ = 0.016). Our polygenic risk score defined on the three genome-wide significant loci (P<5E-8) from HGI7no provided a statistically significant R^2^ = 1.01%. Including additional variants did not improve the PRS fit, regardless of the construction strategy (S11 Table). PRS performance is impacted by the heritability, polygenicity and heterogeneity of the phenotype of interest [58]. In our study, the lower heritability of COVID-19 severity may account for the small portion of variability in hospitalization status explained by our PRS. The degree of polygenicity in COVID-19 severity remains unclear; however, we attempted to address this uncertainty by using an additional PRS method that allows for flexible genetic architectures.

The heritability estimate, PRS, and the colocalization analysis further indicate concordance between HostSeq and HGI, suggesting that the COVID-19 severity loci chr3:4580527 and chr6:41515629 are robust.

## Supporting information

S1 Fig Quality Control (QC) in HostSeq. Flowchart describing the multi-step process of sample and variant QC of joint-called HostSeq data. N = 8,474 / 10,059 samples were retained for genetic analysis. PCA was performed on a subset of variants; these PCs are used as covariates in genetic analysis. HWE was performed on the subset of controls with European ancestry [N = 3,876], and variants with P < 1E-50 were removed from all samples.
(PDF)

S2 Fig PCA projection of HostSeq genomes against reference population. HostSeq genomes were merged with the 1000 Genomes reference set (see Methods of the HostSeq resource paper [1]). First two principal components of this merged data are shown here with HostSeq genomes in black and 1000 Genomes samples colored by their ancestry classification: AFR = African, AMR = Admixed American, EAS = East Asian, SAS = South Asian, EUR = European.
(PDF)

S3 Fig Predicted population admixture and ancestry classification in HostSeq. Each bar represents a genome. Proportion of African, East Asian and European ancestries is determined and genomes classified into 8 groups using GRAF-pop (see Methods). They are further combined into 5 ancestry groups: (i) AFR—African and African-American, (ii) AMR—Latin American Asian and Latin American African, (iii) EAS—Asian-Pacific Islander and East Asian, (iv) SAS—South Asian, and (v) EUR—European. 2% of genomes remain uncategorized.
(PDF)

S4 Fig Genetic distances score of HostSeq genomes. The four genetic distances (GD1-4) scores from GRAF-pop (see Methods) represent distance of each genome from several reference populations, and are used to predict ancestry. Barycentric coordinates of GD1 and GD2 are used to predict admixture proportion of African, East Asian and European ancestries.
(PDF)

S5 Fig Quality of HostSeq genomes. (A) Missing rate < 5% (B) Contamination rate < 3% (C) Mean coverage > 10.
(PDF)

S6 Fig Scree plot of PCA. This plot indicates that the eigenvalues start to plateau around PC7. We used the top seven PCs (PC7 is highlighted in red) as covariates in genetic analysis.
(PDF)

S7 Fig Scatter plots of PCs. Pairwise heatmaps of PC1-PC2, PC3-PC4, and PC5-PC6. No outliers are seen on these pairwise plots.
(PDF)

S8 Fig Distribution of PCs. Stacked histograms for the top seven PCs colored by hospitalization status.
(PDF)

S9 Fig Genetic analysis of HostSeq. Flowchart describing the methods for genetic analysis of HostSeq data [N = 8,474] using regenie and PRSice. Primary GWAS was performed on all samples. Additional stratified GWAS results were obtained to check for heterogeneity within HostSeq. A 2 degrees-of-freedom (d.f.) GxSex test was performed to check the effect of genotype-sex interaction. SKAT-O tests analyzed gene-based effects. HGI7no was constructed by removing overlapping HostSeq samples from HGI7. GWAS results were filtered to remove the GIAB difficult-to-sequence regions and MAF < 5% variants. PRS was constructed using the HGI7no summary statistics.
(PDF)

S10 Fig Distribution of Age. Stacked histogram of age (bin width 10), colored by hospitalization status. This shows association between age and hospitalization.
(PDF)

S11 Fig Comparison of HostSeq allele frequencies with gnomAD. gnomAD allele frequencies of non-Finnish European samples for variants passing quality filters are compared with HostSeq allele frequencies of 100% predicted European samples [N = 1,153]. The heatmap of 27 million variants largely shows concordance between the two sets.
(PDF)

S12 Fig Paired QQ-plots and p-value histograms, stratified by MAF. Left) QQ-plots show the expected and observed -Log10 transformed p-values on the X and Y axes. Right) Paired histograms show p-values binned at width 0.05. Genomic control for each MAF-stratification is: λ = 1.073 for 0 > MAF > 0.05 (first panel), λ = 1.046 for 0.05 > MAF > 0.1 (second panel), λ = 1.048 for 0.1 > MAF > 0.25 (third panel), and λ = 1.048 for 0.25 > MAF > 0.5 (fourth panel).
(PDF)

S13 Fig GWAS results including difficult-to-sequence regions. GWAS of all HostSeq samples passing QC. In the Manhattan plot, Y-axis indicates -Log10 p-values of regenie analysis for variants with MAF > 5%, X-axis indicates chromosomes. Grey horizontal line indicates genome-wide significance level of P < 5E-8. In the corresponding QQ-plot, the X and Y axes indicate expected and observed -Log10 p-values, respectively (genomic control λ = 1.05).
(PDF)

S14 Fig Region plots for the top three novel loci from HostSeq compared with HGI7no. Querying the three regions: a) chr15:54131608, b) chr10:107238146, c) chr3:138353967 in HostSeq (top row in each figure) with HGI7no (bottom row in each figure) shows that these variants are in LD with nearby variants. Plots were generated using myLocusZoom.
(PDF)

S15 Fig Gene-based test results of primary GWAS. Post-GWAS functional analysis of the primary HostSeq GWAS included a gene-based test computed by MAGMA. In the Manhattan plot, Y-axis indicates -Log10 p-values of MAGMA analysis for genes, X-axis indicates chromosomes. Grey horizontal line indicates Bonferroni significance level of P < 2.7E-6. In the corresponding QQ-plot, the X and Y axes indicate expected and observed -Log10 p-values, respectively (genomic control λ = 1.1). The significant hit on chromosome 3 is the MRAS gene with 91 SNPs and P = 3.52E-7.
(PDF)

S16 Fig Meta-analysis results of ancestry-stratified GWAS. SAIGE GWAS of five HostSeq ancestries (EAS, SAS, AFR, AMR and EUR) were meta-analyzed using MR-MEGA [N = 8,272]. In the Manhattan plot, Y-axis indicates -Log10 p-values of MR-MEGA analysis for variants with MAF > 5%, X-axis indicates chromosomes. Variants falling in the GIAB difficult-to-sequence regions have been excluded. Variants missing in any of the ancestry sets did not have a meta-analysis result. Grey horizontal line indicates genome-wide significance level of P < 5E-8. In the corresponding QQ-plot, the X and Y axes indicate expected and observed -Log10 p-values, respectively (genomic control λ = 0.991).
(PDF)

S17 Fig GWAS testing the G x Sex interaction effect. The p-values are derived from a 2 degrees-of-freedom test that considers both genotype, and interaction between genotype and sex jointly. In the Manhattan plot, Y-axis indicates -Log10 p-values of regenie analysis for variants with MAF > 5%, X-axis indicates chromosomes. Variants falling in the GIAB difficult-to-sequence regions have been excluded. Grey horizontal line indicates genome-wide significance level of P < 5E-8. In the corresponding QQ-plot, the X and Y axes indicate expected and observed -Log10 p-values, respectively (genomic control λ = 1.194).
(PDF)

S18 Fig Region plot for the top novel locus identified through the G x Sex interaction test compared with other results. Querying the chr7:107127037 region in G x Sex GWAS (top row) shows that this variant is in LD with nearby variants. Comparing it with the following in order: primary GWAS, sex-stratified GWAS for males, sex-stratified GWAS for females, and HGI7no shows that there is a sex-effect for this locus in HostSeq, where males [N = 3,646] have an association with hospitalization. Plots were generated using myLocusZoom.
(PDF)

S19 Fig SKAT-O results for gene-based testing including rare variants. Top) Manhattan plot for the high impact set (3,350 genes). Middle) Manhattan plot for the high/moderate impact set (17,341 genes). Bottom) QQ-plots for the high impact (bottom left), and high/moderate impact (bottom right) sets. SKAT-O analysis was performed on variants outside the GIAB difficult-to-sequence regions.
(PDF)

S1 Table Summary statistics per ancestry. Samples were assigned ancestry based on prediction by GRAF-pop (see Methods), and then categorized into 5 superpopulations: AFR = African, AMR = Admixed American, EAS = East Asian, SAS = South Asian, EUR = European. EUR is the largest ancestry in HostSeq.
(XLSX)

S2 Table Summary statistics per study HostSeq constitutes several studies of varying sizes and hostpitalization proportions. Some studies share samples, however, in this table overlapping samples have been only been counted once. BQC19 is the largest study in HostSeq. GenOMICC has the highest proportion of hospitalized samples.
(XLSX)

S3 Table Per-gene association details from the MAGMA gene-set analysis. The significant gene-set ‘HASEGAWA_TUMORIGENESIS_BY_RET_C634R’ has an effect size of 1.72+/-0.31 and a raw p-value of 2.36E-8.
(XLSX)

S4 Table Association details of 47 variants from the HGI GWAS comparing HGI7no results with HostSeq. Direction and magnitude of effect size is consistent between HGI7no and HostSeq for most of the loci. Nearest-Gene and Suggested-Phenotype annotations are as provided by HGI (table S2 of HGI 2023 Nature paper) [8]. Suggested-phenotype indicates the result of HGI’s phenotypic impact assessment to determine if ‘disease severity’ or ‘infection susceptibility’ is the main impact.
(XLSX)

S5 Table Ancestry-stratified allele frequencies for HGI7no hits in HostSeq. Columns labeled by ancestry codes indicate the effect allele frequency for each variant in HostSeq ancestries.
(XLSX)

S6 Table Study-stratified allele frequencies for HGI7no hits in HostSeq. Columns labeled by study acronyms indicate the effect allele frequency for each variant in HostSeq studies.
(XLSX)

S7 Table Association details of lead variant from HostSeq G x Sex interaction analysis. Variant rs79973703 (at the GRCh38 genomic location of chr7:107127037) was identified from a joint 2 d.f. test after applying a MAF > 5% filter and removing variants in the difficult-to-sequence regions. Top row indicates results from the primary single-variant analysis. Following two rows indicate sex-stratified single-variant results. Subsequent rows indicate results from the G x Sex interaction GWAS.
(XLSX)

S8 Table Association details of variants in WDR89. WDR89 (at the GRCh38 genomic location of chr14:63597039–63641871) was identified from a SKAT-O analysis of high/moderate impact variants. 7 missense variants in WDR89 have a result in the primary unfiltered GWAS. Amino Acid change is depicted in the HGVS (Human Genome Variation Society) notation.
(XLSX)

S9 Table Quality metrics for selected HostSeq variants. Variants were selected to include the top three HGI7no loci (first three rows), the top five hits from the primary HostSeq GWAS (next five rows), and the three WDR89 variants driving the SKAT-O results (last three rows). Values have been extracted from the joint-called VCF of N = 10,059 samples before QC removals (these variants passed all filters described in S1 Fig). WDR89 variants have the lowest ‘MAF’ and higher ‘ExcessHet’ but they pass HostSeq thresholds. However, they failed in gnomAD (v4.0) which used a different method, AS_VQSR, for QC protocol. Description of column headers is given below the table.
(XLSX)

S10 Table Association of PRS with hospitalization status. PRS is constructed with PRSice using the top three loci which pass the P < 5E-8 threshold in HGI7no. Association results in this table exclude genetic PCs as covariates from the model. While an examination of PRS without genetic PCs is significant, inclusion of genetic PCs reduces significance from P = 1.96E-48 (Table 5) to 5.25E-13. Retained signal indicates that population structure does not confound our analysis.
(XLSX)

S11 Table Association between PRS and hospitalization status by different methods. P-value thresholds are indicated for PRSice results, and results are compared to PRS-CS method (last row). PRSice thresholds at P < 5E-8 and 1E-5 are significant at 0.05 level and include a small number of variants indicating low polygenicity (at most 53, after LD clumping with a window-size of 750kb). PRS-CS result is also significant but uses a large number of SNPs. All significant thresholds have positive effect size, indicating that polygenic risk for hospitalization calculated with summary statistics from HGI7no is positively associated with hospitalization in HostSeq. R-squared represents difference in pseudo-R-squared of full model with PRS and null model without PRS.
(XLSX)

S12 Table Per-ancestry association of PRS with hospitalization status. PRS is constructed with (a) PRSice using the top three loci which pass the P < 5E-8 threshold in HGI7no, and (b) PRS-CS using 1,033,441 SNPs. Association is tested per-ancestry using the same model as in S9 Table, i.e., with all covariates except genetic PCs. Beta, SE, t-statistic and p-value is reported for the PRS term in the table for all ancestries. R-squared represents difference in pseudo-R-squared of full model with PRS and null model without PRS.
(XLSX)
