# Correction: Canadian COVID-19 host genetics cohort replicates known severity associations

**DOI:** 10.1371/journal.pgen.1011628

**Published:** 2025-03-04

**Authors:** Elika Garg, Paola Arguello-Pascualli, Olga Vishnyakova, Anat R. Halevy, Samantha Yoo, Jennifer D. Brooks, Shelley B. Bull, France Gagnon, Celia M. T. Greenwood, Rayjean J. Hung, Jerald F. Lawless, Jordan Lerner-Ellis, Jessica K. Dennis, Rohan J. S. Abraham, Jean-Michel Garant, Bhooma Thiruvahindrapuram, Steven J. M. Jones, CGEn HostSeq Initiative, Lisa J. Strug, Andrew D. Paterson, Lei Sun, Lloyd T. Elliott

There are errors in the Funding statement. The correct Funding statement is as follows: ADP and LS are supported by the Canadian Institutes of Health Research Project Grant 470360 (https://cihr-irsc.gc.ca). LJS is supported by the Canadian Institutes of Health Research Foundation Grant 167282 (https://cihr-irsc.gc.ca) and Canada Research Chairs (https://www.chairs-chaires.gc.ca/). JL-E is supported by the Canadian Institutes of Health Research Foundation Grant VR4-172753 (https://cihr-irsc.gc.ca). LTE is supported by the Michael Smith Health Research BC Scholar Award SCH-2022-2784 (https://healthresearchbc.ca), and CGEn (Canada’s National Platform for Genome Sequencing and Analysis). The funders have no role in study design, data collection and analysis, decision to publish, or preparation of the manuscript.

There are errors in the Acknowledgement section. The correct Acknowledgement section is as follows: This research has been conducted using CGEn’s HostSeq Databank funded by the Government of Canada through Genome Canada under Project ID DACO-2. We wish to express gratitude to all HostSeq project participant studies and the individual participants within these studies for their contribution. We would also like to thank Natalie Sun, Samantha Roper and Charlene Bradbury for help with administration.
