# Supplementary material for: Canadian COVID-19 host genetics cohort replicates known severity associations
Source: PLoS Genet. 2024 Mar 22;20(3):e1011192. doi: 10.1371/journal.pgen.1011192 (PMC10990181; doi:10.1371/journal.pgen.1011192)
Supplement: S1 Fig — Flowchart describing the multi-step process of sample and variant QC of joint-called HostSeq data. N = 8,474 / 10,059 samples were retained for genetic analysis. PCA was performed on a subset of variants; these PCs are used as covariates in genetic analysis. HWE was performed on the subset of controls with European ancestry [N = 3,876], and variants with P < 1E-50 were removed from all samples. (PDF) [file pgen.1011192.s001.pdf]

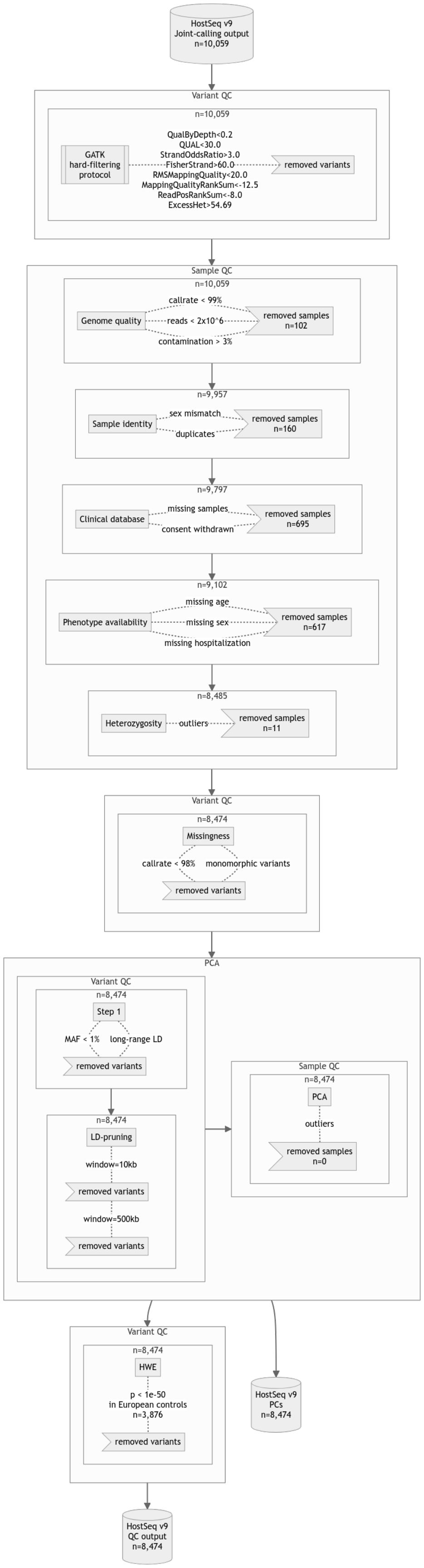
HostSeq v9
Joint-calling output
n=10,059
Variant QC
n=10,059
GATK
hard-filtering
protocol
QualByDepth<0.2
QUAL<30.0
StrandOddsRatio>3.0
FisherStrand>60.0
RMSMappingQuality<20.0
MappingQualityRankSum<-12.5
ReadPosRankSum<-8.0
ExcessHet>54.69
removed variants
Sample QC
n=10,059
Genome quality
callrate < 99%
reads < 2x10^6
contamination > 3%
removed samples
n=102
n=9,957
Sample identity
sex mismatch
duplicates
removed samples
n=160
n=9,797
Clinical database
missing samples
consent withdrawn
removed samples
n=695
n=9,102
Phenotype availability
missing age
missing sex
missing hospitalization
removed samples
n=617
n=8,485
Heterozygosity
outliers
removed samples
n=11
Variant QC
n=8,474
Missingness
callrate < 98%
monomorphic variants
removed variants
PCA
Variant QC
n=8,474
Step 1
MAF < 1%
long-range LD
removed variants
n=8,474
LD-pruning
window=10kb
removed variants
window=500kb
removed variants
Sample QC
n=8,474
PCA
outliers
removed samples
n=0
Variant QC
n=8,474
HWE
p < 1e-50
in European controls
n=3,876
removed variants
HostSeq v9
PCs
n=8,474
HostSeq v9
QC output
n=8,474

Figure S1. Quality Control (QC) in HostSeq. Flowchart describing the multi-step process of sample and variant QC of joint-called HostSeq data. N = 8,474 / 10,059 samples were retained for genetic analysis. PCA was performed on a subset of variants; these PCs are used as covariates in genetic analysis. HWE was performed on the subset of controls with European ancestry [N = 3,876], and variants with P < 1E-50 were removed from all samples.
