# Supplementary material for: Canadian COVID-19 host genetics cohort replicates known severity associations
Source: PLoS Genet. 2024 Mar 22;20(3):e1011192. doi: 10.1371/journal.pgen.1011192 (PMC10990181; doi:10.1371/journal.pgen.1011192)
Supplement: S2 Fig — HostSeq genomes were merged with the 1000 Genomes reference set (see Methods of the HostSeq resource paper [1]). First two principal components of this merged data are shown here with HostSeq genomes in black and 1000 Genomes samples colored by their ancestry classification: AFR = African, AMR = Admixed American, EAS = East Asian, SAS = South Asian, EUR = European. (PDF) [file pgen.1011192.s002.pdf]

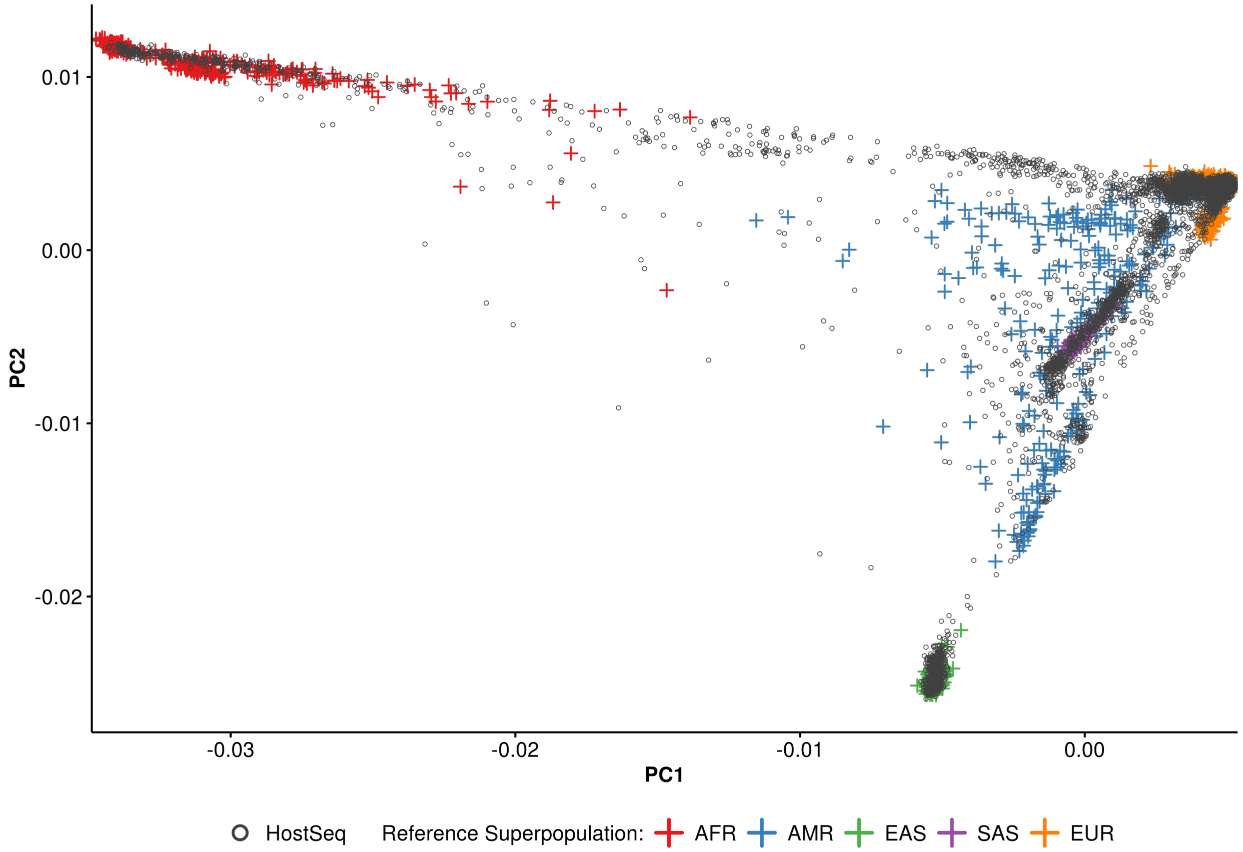

Figure S2. PCA projection of HostSeq genomes against reference population. HostSeq genomes were merged with the 1000 Genomes reference set (see Methods of the HostSeq resource paper (1)). First two principal components of this merged data are shown here with HostSeq genomes in black and 1000 Genomes samples colored by their ancestry classification: AFR=African, AMR=Admixed American, EAS=East Asian, SAS=South Asian, EUR=European.
