# Supplementary material for: Canadian COVID-19 host genetics cohort replicates known severity associations
Source: PLoS Genet. 2024 Mar 22;20(3):e1011192. doi: 10.1371/journal.pgen.1011192 (PMC10990181; doi:10.1371/journal.pgen.1011192)
Supplement: S3 Fig — Each bar represents a genome. Proportion of African, East Asian and European ancestries is determined and genomes classified into 8 groups using GRAF-pop (see Methods). They are further combined into 5 ancestry groups: (i) AFR—African and African-American, (ii) AMR—Latin American Asian and Latin American African, (iii) EAS—Asian-Pacific Islander and East Asian, (iv) SAS—South Asian, and (v) EUR—European. 2% of genomes remain uncategorized. (PDF) [file pgen.1011192.s003.pdf]

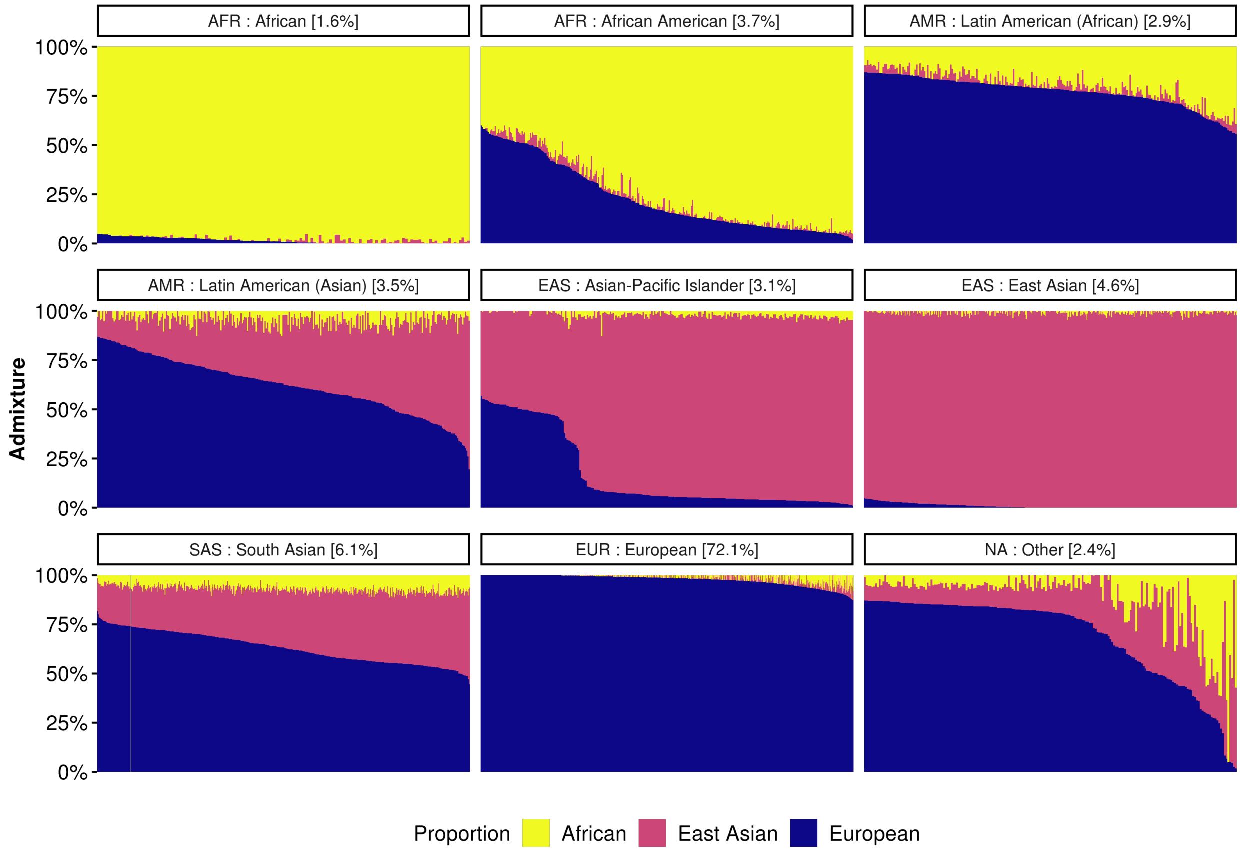

Figure S3. Predicted population admixture and ancestry classification in HostSeq. Each bar represents a genome. Proportion of African, East Asian and European ancestries is determined and genomes classified into 8 groups using GRAF-pop (see Methods). They are further combined into 5 ancestry groups : (i) AFR - African and African-American, (ii) AMR - Latin American Asian and Latin American African, (iii) EAS - Asian-Pacific Islander and East Asian, (iv) SAS - South Asian, and (v) EUR - European. 2% of genomes remain uncategorized.
