# Supplementary material for: Canadian COVID-19 host genetics cohort replicates known severity associations
Source: PLoS Genet. 2024 Mar 22;20(3):e1011192. doi: 10.1371/journal.pgen.1011192 (PMC10990181; doi:10.1371/journal.pgen.1011192)
Supplement: S4 Fig — The four genetic distances (GD1-4) scores from GRAF-pop (see Methods) represent distance of each genome from several reference populations, and are used to predict ancestry. Barycentric coordinates of GD1 and GD2 are used to predict admixture proportion of African, East Asian and European ancestries. (PDF) [file pgen.1011192.s004.pdf]

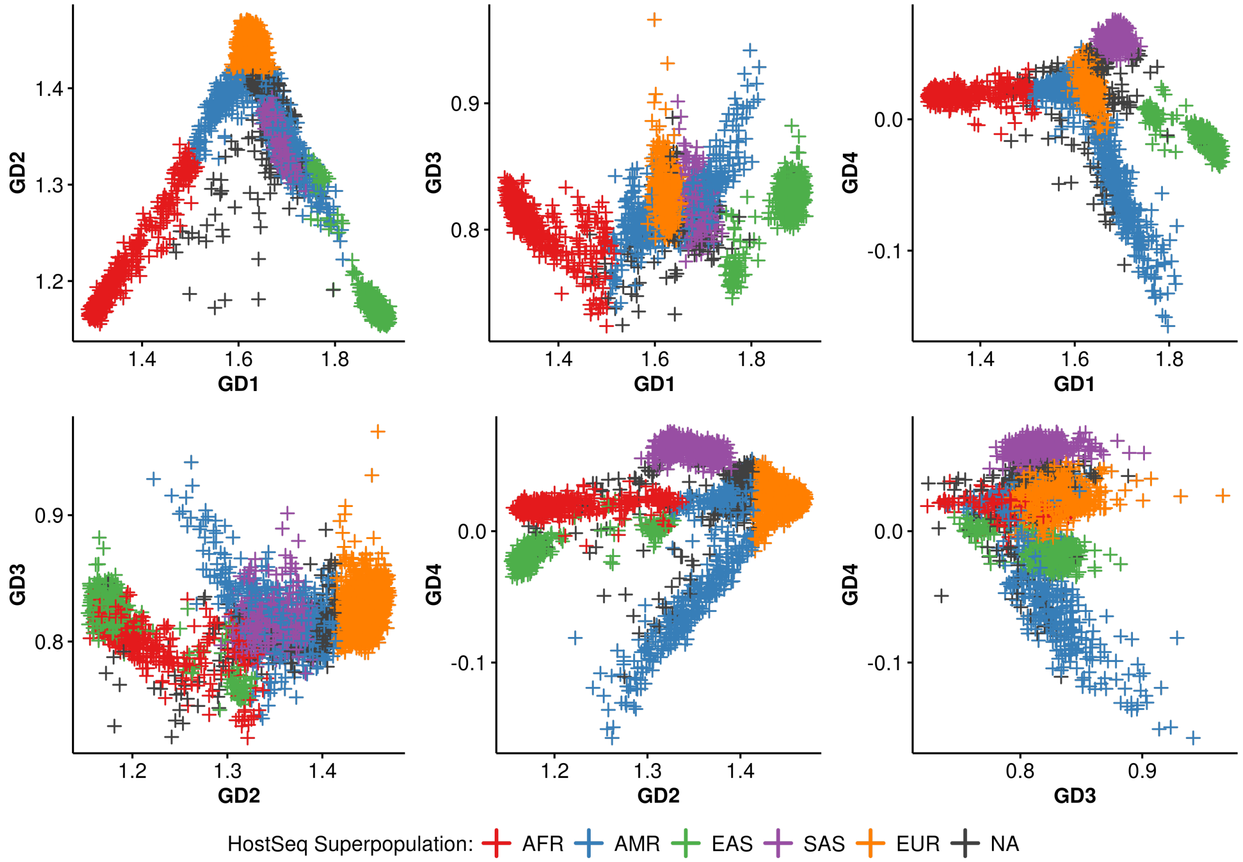

Figure S4. Genetic distances score of HostSeq genomes. The four genetic distances (GD1-4) scores from GRAF-pop (see Methods) represent distance of each genome from several reference populations, and are used to predict ancestry. Barycentric coordinates of GD1 and GD2 are used to predict admixture proportion of African, East Asian and European ancestries.
