# Supplementary figures and images for: Canadian COVID-19 host genetics cohort replicates known severity associations
Source: PLoS Genet. 2024 Mar 22;20(3):e1011192. doi: 10.1371/journal.pgen.1011192 (PMC10990181; doi:10.1371/journal.pgen.1011192)

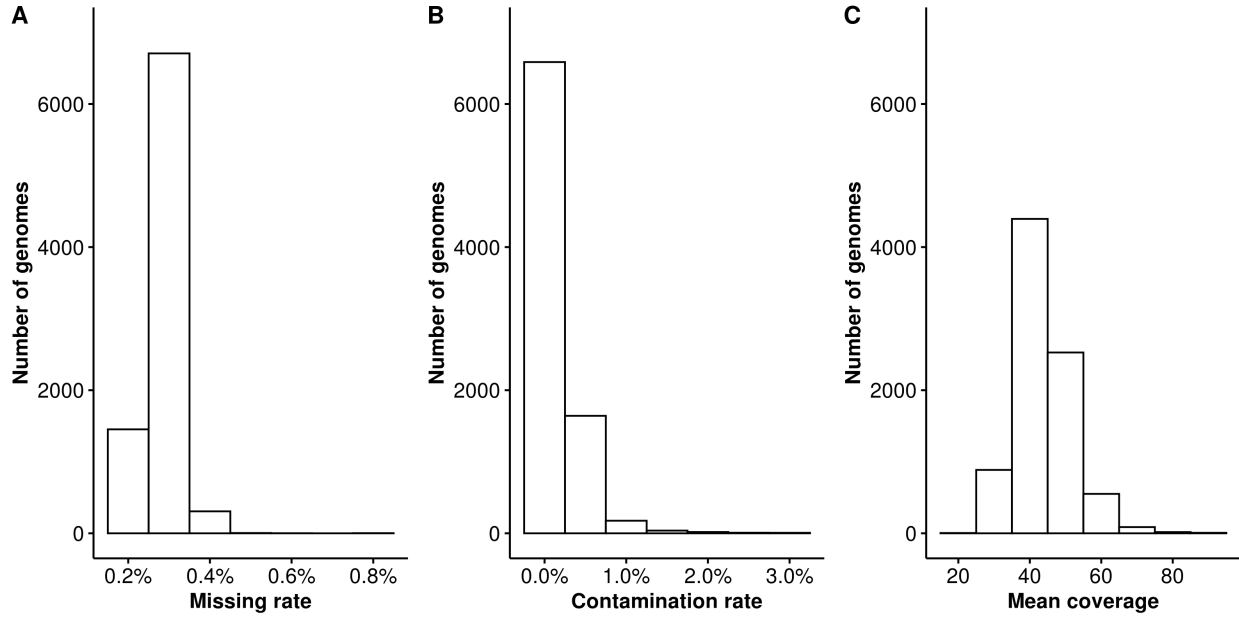

**Figure S5. Quality of HostSeq genomes.** (A) Missing rate < 5% (B) Contamination rate < 3% (C) Mean coverage > 10.

Supplement: S5 Fig — (A) Missing rate < 5% (B) Contamination rate < 3% (C) Mean coverage > 10. (PDF) [file pgen.1011192.s005.pdf]
