# Supplementary material for: Canadian COVID-19 host genetics cohort replicates known severity associations
Source: PLoS Genet. 2024 Mar 22;20(3):e1011192. doi: 10.1371/journal.pgen.1011192 (PMC10990181; doi:10.1371/journal.pgen.1011192)
Supplement: S6 Fig — This plot indicates that the eigenvalues start to plateau around PC7. We used the top seven PCs (PC7 is highlighted in red) as covariates in genetic analysis. (PDF) [file pgen.1011192.s006.pdf]

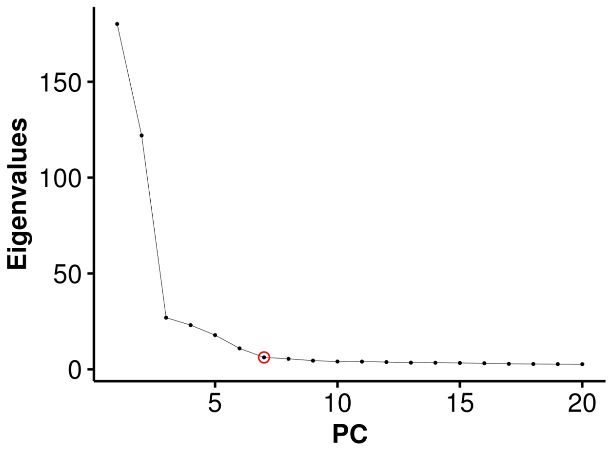

Figure S6. Scree plot of PCA. This plot indicates that the eigenvalues start to plateau around PC7. We used the top seven PCs (PC7 is highlighted in red) as covariates in genetic analysis.
