# Supplementary material for: Canadian COVID-19 host genetics cohort replicates known severity associations
Source: PLoS Genet. 2024 Mar 22;20(3):e1011192. doi: 10.1371/journal.pgen.1011192 (PMC10990181; doi:10.1371/journal.pgen.1011192)
Supplement: S7 Fig — Pairwise heatmaps of PC1-PC2, PC3-PC4, and PC5-PC6. No outliers are seen on these pairwise plots. (PDF) [file pgen.1011192.s007.pdf]

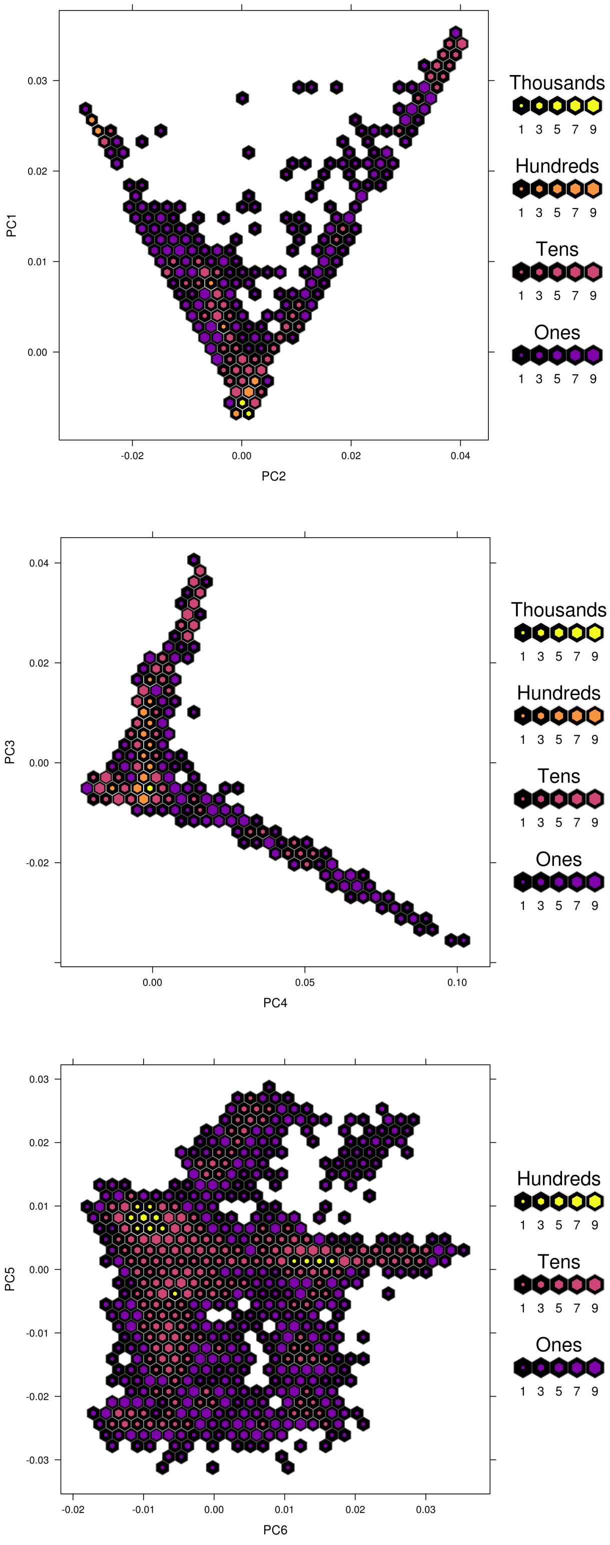

Figure S7. Scatter plots of PCs. Pairwise heatmaps of PC1-PC2, PC3-PC4, and PC5-PC6. No outliers are seen on these pairwise plots.
