# Supplementary material for: Canadian COVID-19 host genetics cohort replicates known severity associations
Source: PLoS Genet. 2024 Mar 22;20(3):e1011192. doi: 10.1371/journal.pgen.1011192 (PMC10990181; doi:10.1371/journal.pgen.1011192)
Supplement: S8 Fig — Stacked histograms for the top seven PCs colored by hospitalization status. (PDF) [file pgen.1011192.s008.pdf]

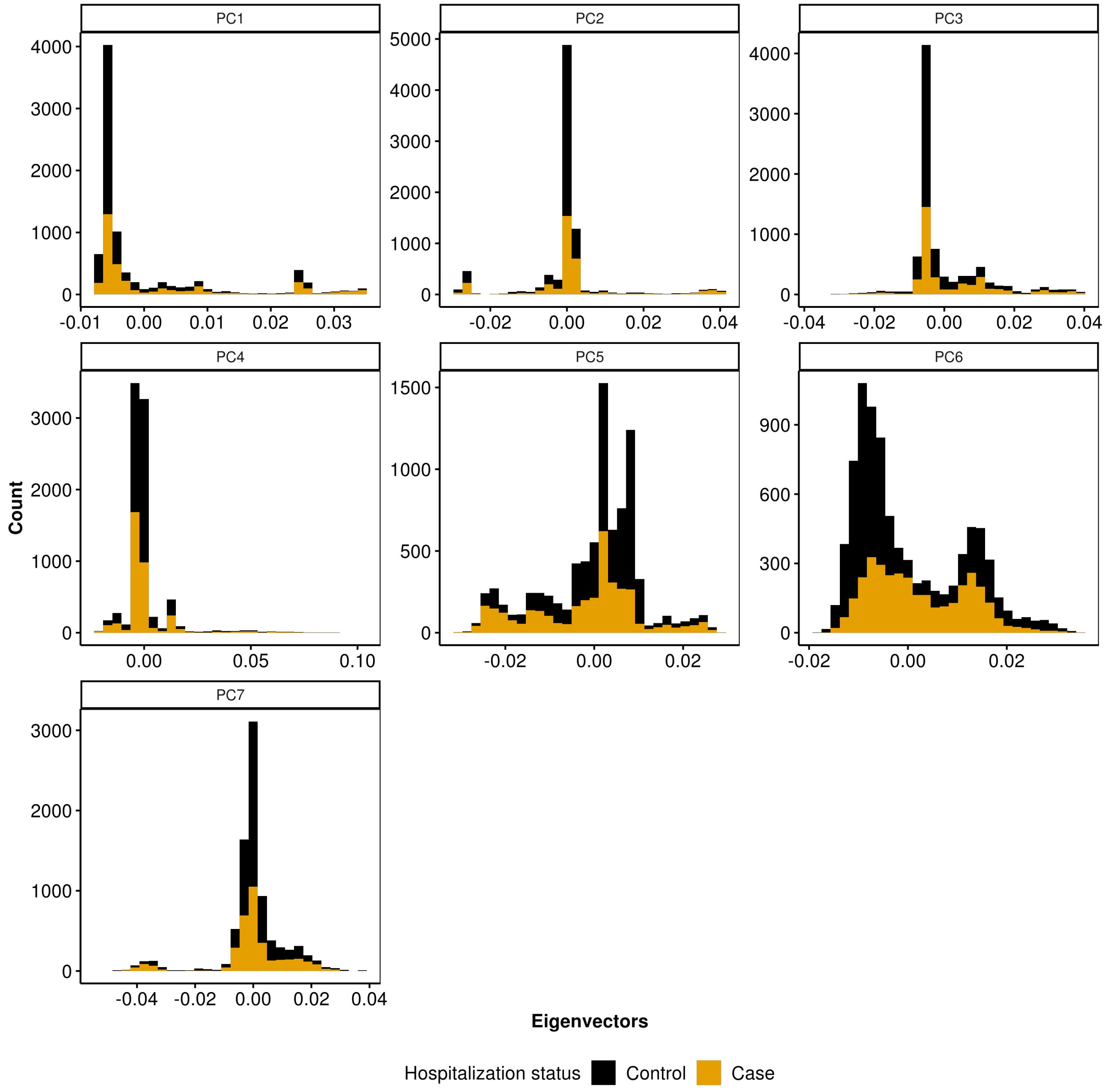

Figure S8. Distribution of PCs. Stacked histograms for the top seven PCs colored by hospitalization status.
