# Supplementary material for: Canadian COVID-19 host genetics cohort replicates known severity associations
Source: PLoS Genet. 2024 Mar 22;20(3):e1011192. doi: 10.1371/journal.pgen.1011192 (PMC10990181; doi:10.1371/journal.pgen.1011192)
Supplement: S9 Fig — Flowchart describing the methods for genetic analysis of HostSeq data [N = 8,474] using regenie and PRSice. Primary GWAS was performed on all samples. Additional stratified GWAS results were obtained to check for heterogeneity within HostSeq. A 2 degrees-of-freedom (d.f.) GxSex test was performed to check the effect of genotype-sex interaction. SKAT-O tests analyzed gene-based effects. HGI7no was constructed by removing overlapping HostSeq samples from HGI7. GWAS results were filtered to remove the GIAB difficult-to-sequence regions and MAF < 5% variants. PRS was constructed using the HGI7no summary statistics. (PDF) [file pgen.1011192.s009.pdf]

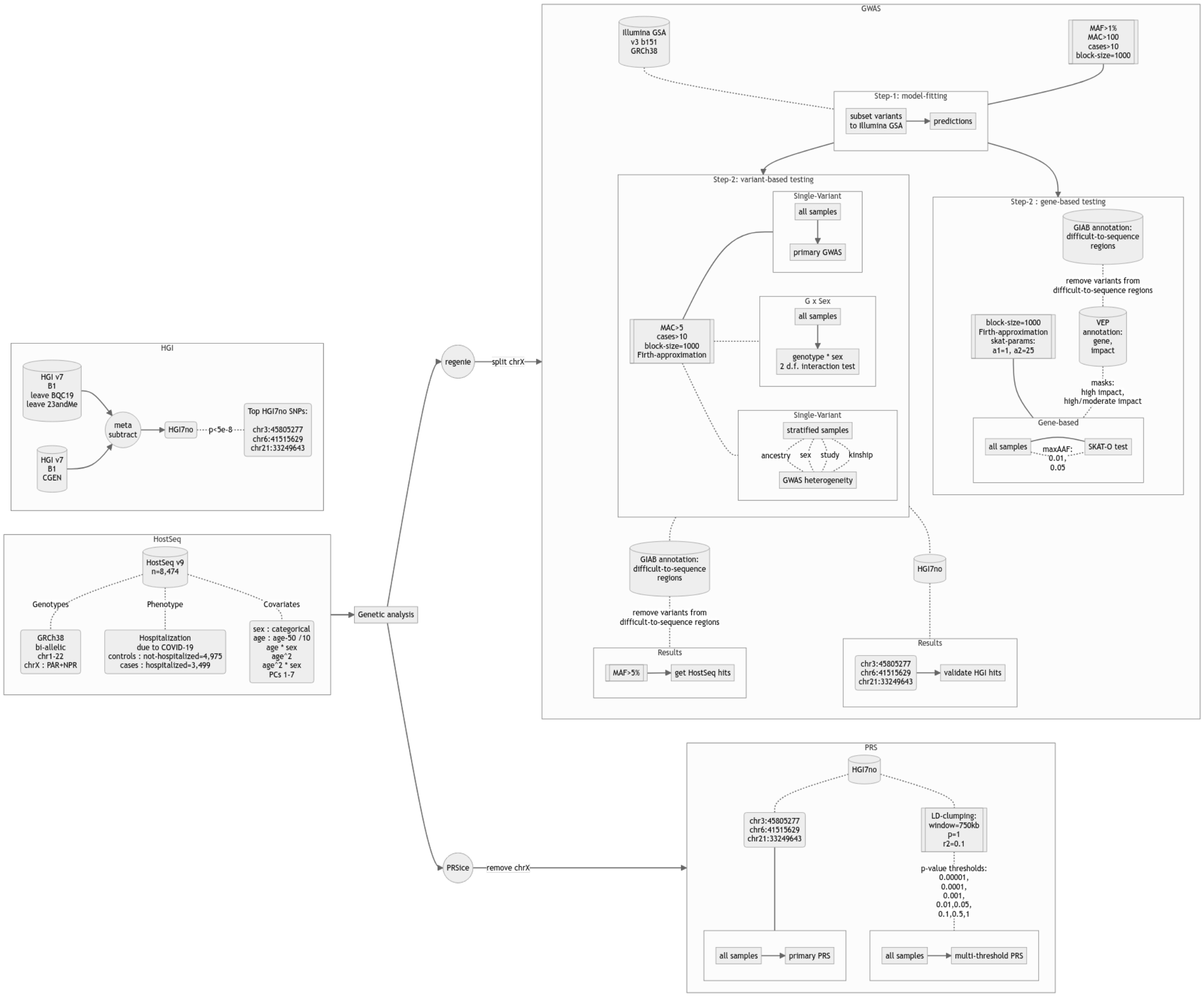

Figure S9. Genetic analysis of HostSeq. Flowchart describing the methods for genetic analysis of HostSeq data [N = 8,474] using regenie and PRSice. Primary GWAS was performed on all samples. Additional stratified GWAS results were obtained to check for heterogeneity within HostSeq. A 2 degrees-of-freedom (d.f.) GxSex test was performed to check the effect of genotype-sex interaction. SKAT-O tests analyzed gene-based effects. HGI7no was constructed by removing overlapping HostSeq samples from HGI7. GWAS results were filtered to remove the GIAB difficult-to-sequence regions and MAF $< 5\%$ variants. PRS was constructed using the HGI7no summary statistics.
