# Supplementary material for: Canadian COVID-19 host genetics cohort replicates known severity associations
Source: PLoS Genet. 2024 Mar 22;20(3):e1011192. doi: 10.1371/journal.pgen.1011192 (PMC10990181; doi:10.1371/journal.pgen.1011192)
Supplement: S10 Fig — Stacked histogram of age (bin width 10), colored by hospitalization status. This shows association between age and hospitalization. (PDF) [file pgen.1011192.s010.pdf]

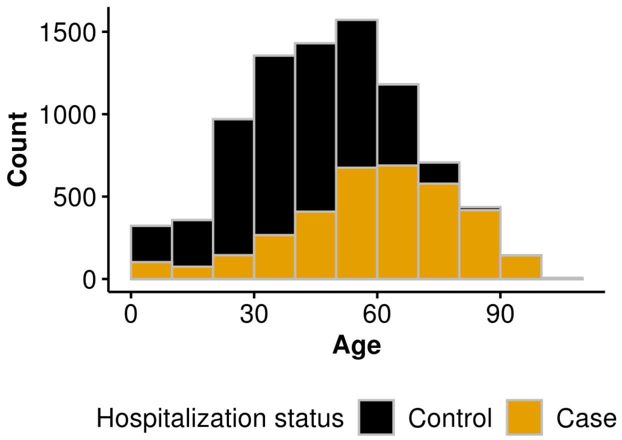

Figure S10. Distribution of Age. Stacked histogram of age (bin width 10), colored by hospitalization status. This shows association between age and hospitalization.
