# Supplementary material for: Canadian COVID-19 host genetics cohort replicates known severity associations
Source: PLoS Genet. 2024 Mar 22;20(3):e1011192. doi: 10.1371/journal.pgen.1011192 (PMC10990181; doi:10.1371/journal.pgen.1011192)
Supplement: S11 Fig — gnomAD allele frequencies of non-Finnish European samples for variants passing quality filters are compared with HostSeq allele frequencies of 100% predicted European samples [N = 1,153]. The heatmap of 27 million variants largely shows concordance between the two sets. (PDF) [file pgen.1011192.s011.pdf]

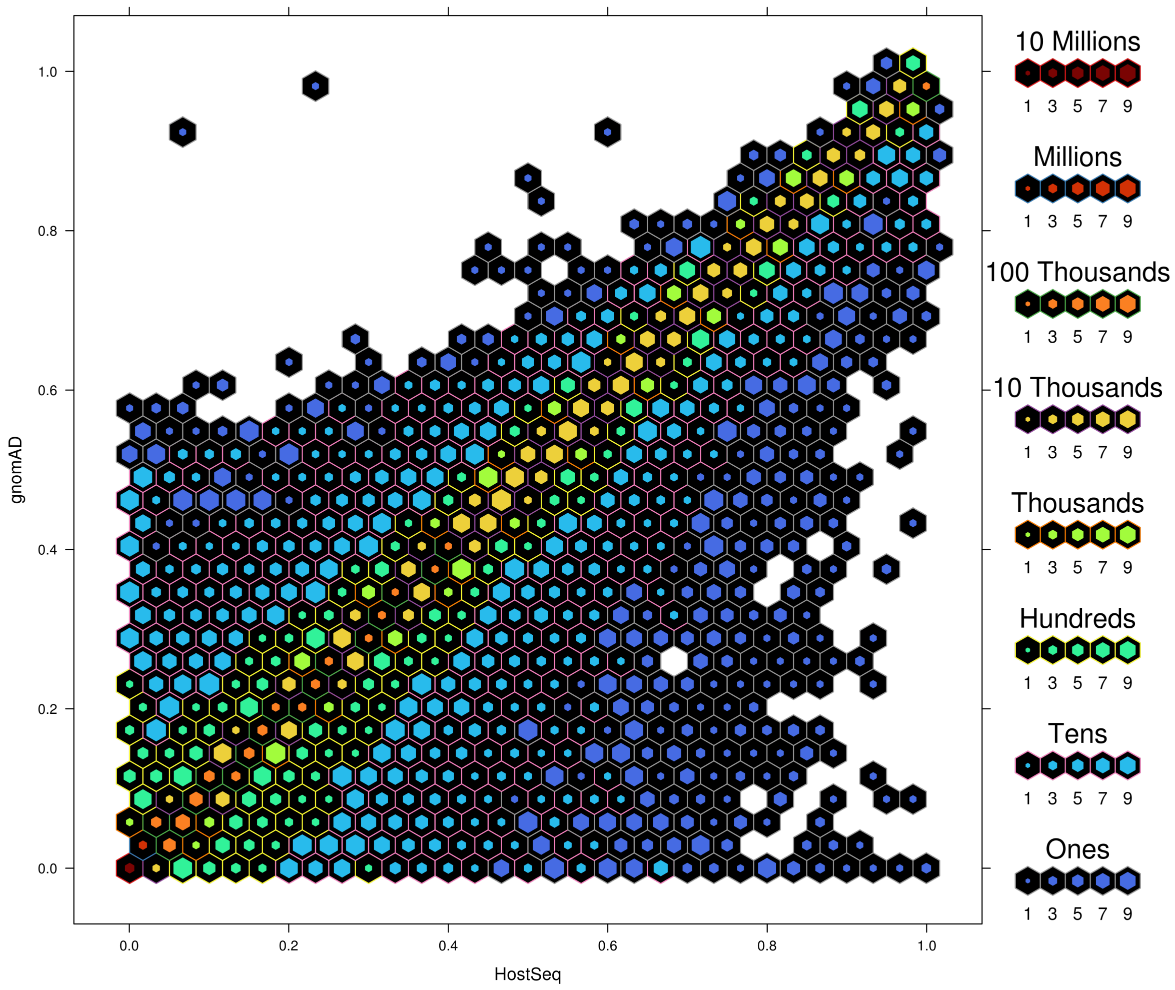

Figure S11. Comparison of HostSeq allele frequencies with gnomAD. gnomAD allele frequencies of non-Finnish European samples for variants passing quality filters are compared with HostSeq allele frequencies of 100% predicted European samples [N = 1,153]. The heatmap of 27 million variants largely shows concordance between the two sets.
