# Supplementary material for: Canadian COVID-19 host genetics cohort replicates known severity associations
Source: PLoS Genet. 2024 Mar 22;20(3):e1011192. doi: 10.1371/journal.pgen.1011192 (PMC10990181; doi:10.1371/journal.pgen.1011192)
Supplement: S12 Fig — Left) QQ-plots show the expected and observed -Log10 transformed p-values on the X and Y axes. Right) Paired histograms show p-values binned at width 0.05. Genomic control for each MAF-stratification is: λ = 1.073 for 0 > MAF > 0.05 (first panel), λ = 1.046 for 0.05 > MAF > 0.1 (second panel), λ = 1.048 for 0.1 > MAF > 0.25 (third panel), and λ = 1.048 for 0.25 > MAF > 0.5 (fourth panel). (PDF) [file pgen.1011192.s012.pdf]

0 < MAF < 0.05

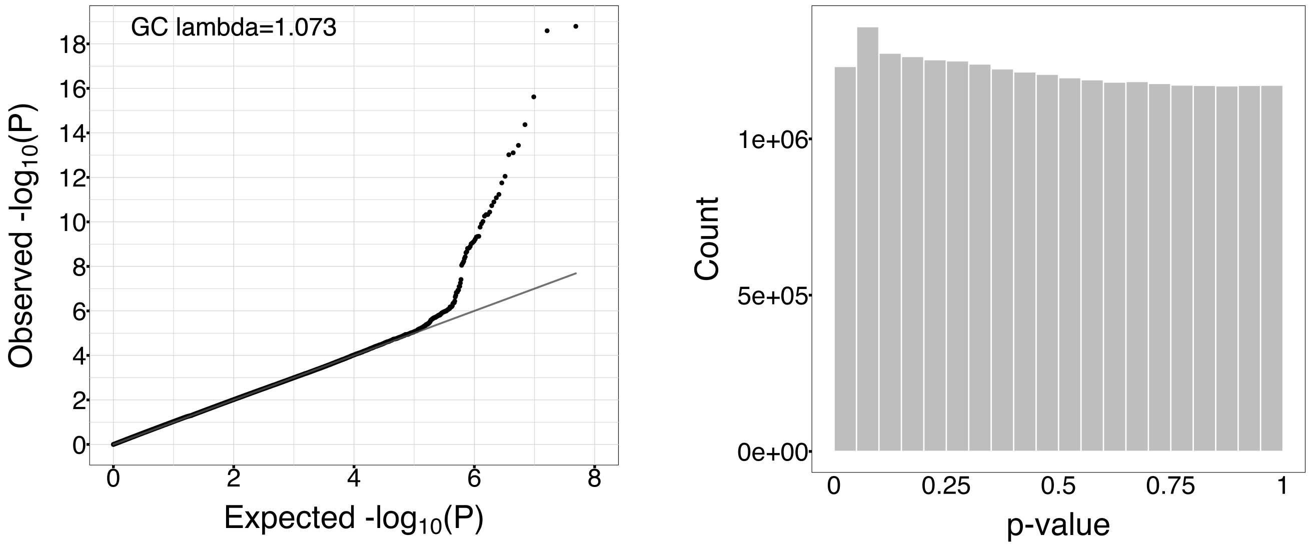

0.05 < MAF < 0.1

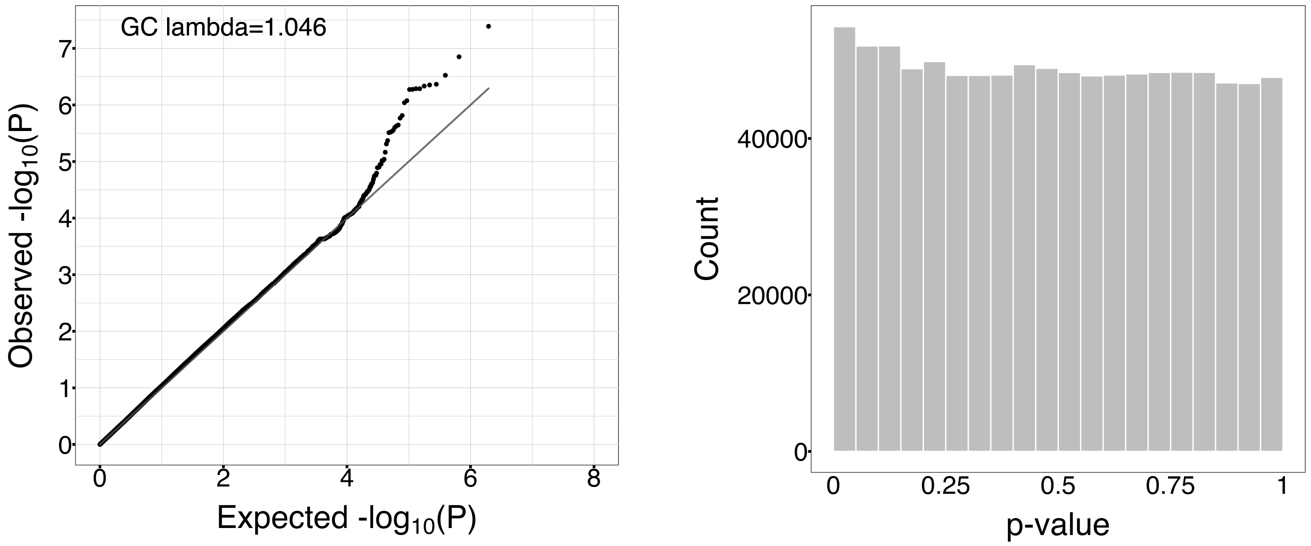

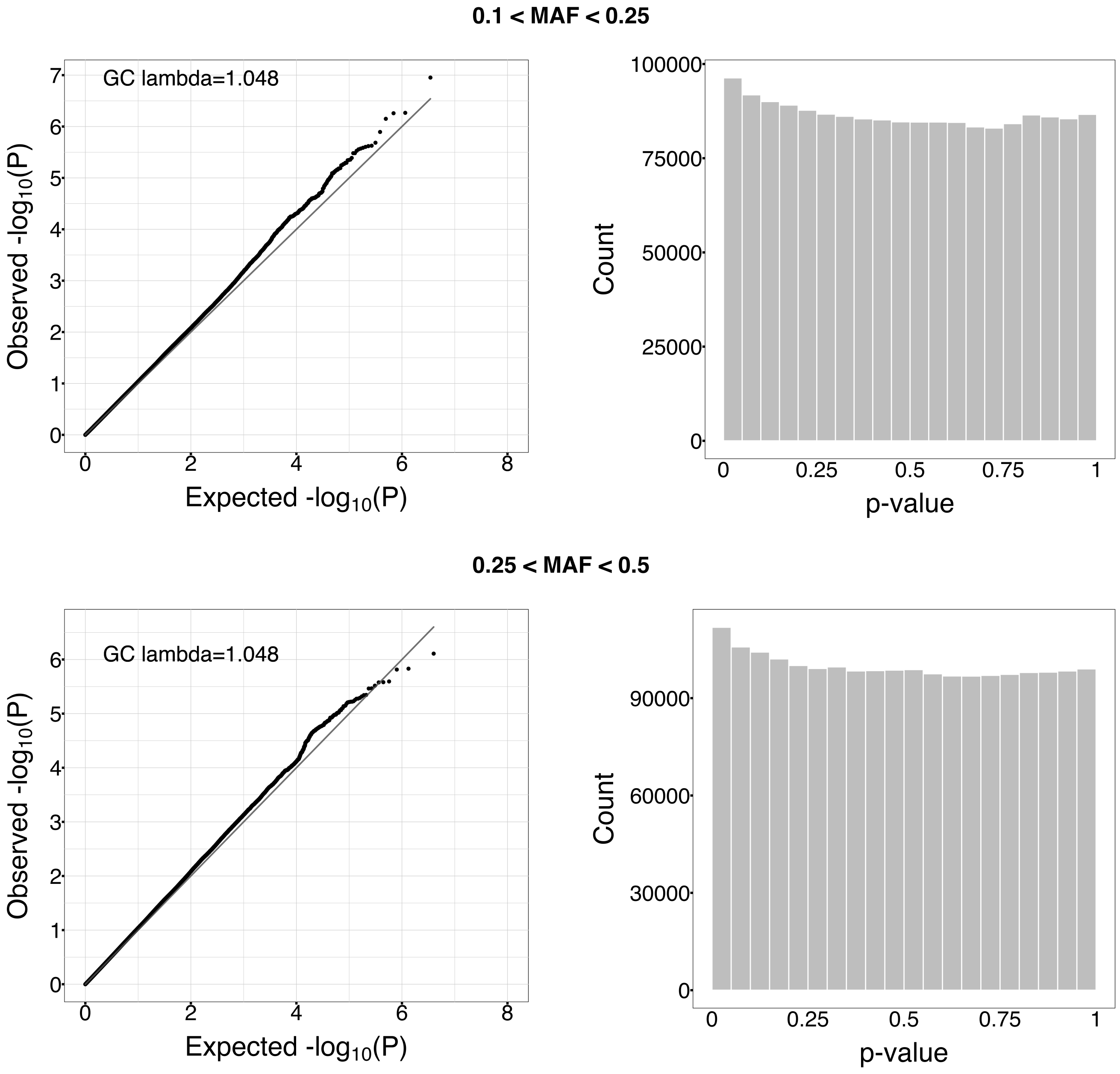

Figure S12. Paired QQ-plots and p-value histograms, stratified by MAF. Left) QQ-plots show the expected and observed -Log10 transformed p-values on the X and Y axes. Right) Paired histograms show p-values binned at width 0.05. Genomic control for each MAF-stratification is: $\lambda = 1.073$ for $0 > \text{MAF} > 0.05$ (first panel), $\lambda = 1.046$ for $0.05 > \text{MAF} > 0.1$ (second panel), $\lambda = 1.048$ for $0.1 > \text{MAF} > 0.25$ (third panel), and $\lambda = 1.048$ for $0.25 > \text{MAF} > 0.5$ (fourth panel).
