# Supplementary material for: Canadian COVID-19 host genetics cohort replicates known severity associations
Source: PLoS Genet. 2024 Mar 22;20(3):e1011192. doi: 10.1371/journal.pgen.1011192 (PMC10990181; doi:10.1371/journal.pgen.1011192)
Supplement: S13 Fig — GWAS of all HostSeq samples passing QC. In the Manhattan plot, Y-axis indicates -Log10 p-values of regenie analysis for variants with MAF > 5%, X-axis indicates chromosomes. Grey horizontal line indicates genome-wide significance level of P < 5E-8. In the corresponding QQ-plot, the X and Y axes indicate expected and observed -Log10 p-values, respectively (genomic control λ = 1.05). (PDF) [file pgen.1011192.s013.pdf]

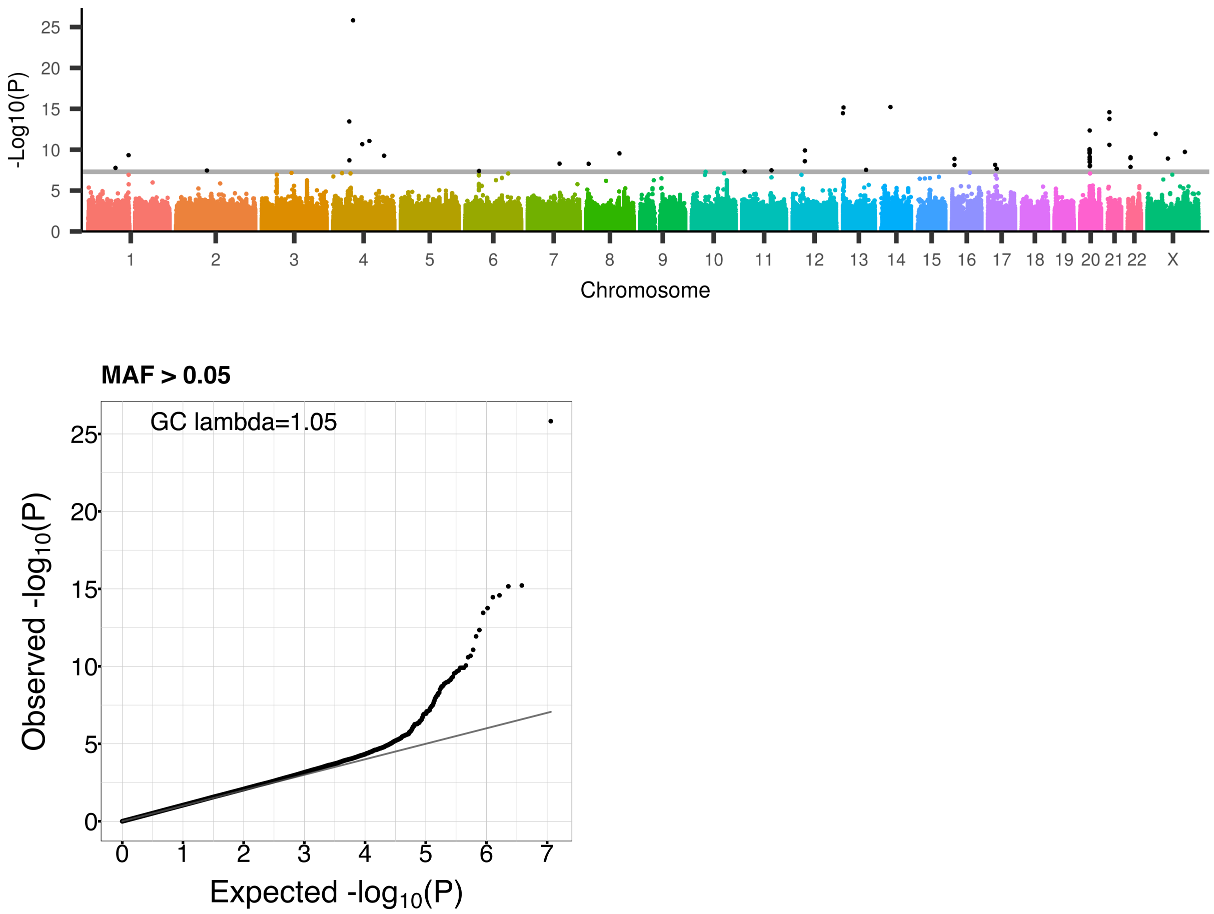

Figure S13. GWAS results including difficult-to-sequence regions. GWAS of all HostSeq samples passing QC. In the Manhattan plot, Y-axis indicates -Log10 p-values of regenie analysis for variants with MAF > 5%, X-axis indicates chromosomes. Grey horizontal line indicates genome-wide significance level of P < 5E-8. In the corresponding QQ-plot, the X and Y axes indicate expected and observed -Log10 p-values, respectively (genomic control $\lambda = 1.05$).
