# Supplementary material for: Canadian COVID-19 host genetics cohort replicates known severity associations
Source: PLoS Genet. 2024 Mar 22;20(3):e1011192. doi: 10.1371/journal.pgen.1011192 (PMC10990181; doi:10.1371/journal.pgen.1011192)
Supplement: S14 Fig — Querying the three regions: a) chr15:54131608, b) chr10:107238146, c) chr3:138353967 in HostSeq (top row in each figure) with HGI7no (bottom row in each figure) shows that these variants are in LD with nearby variants. Plots were generated using myLocusZoom. (PDF) [file pgen.1011192.s014.pdf]

a)

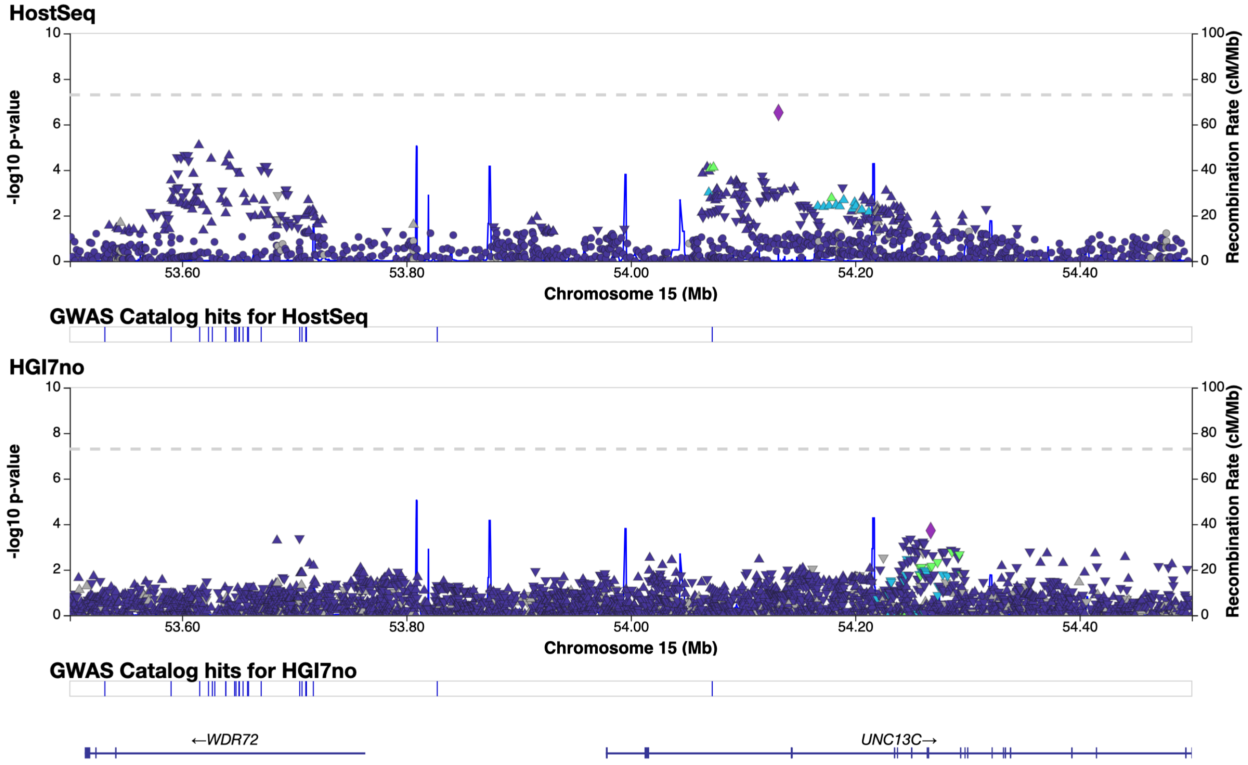
HostSeq
-log10 p-value
Recombination Rate (cM/Mb)
Chromosome 15 (Mb)
GWAS Catalog hits for HostSeq
HGI7no
GWAS Catalog hits for HGI7no
←WDR72
UNC13C→

b)

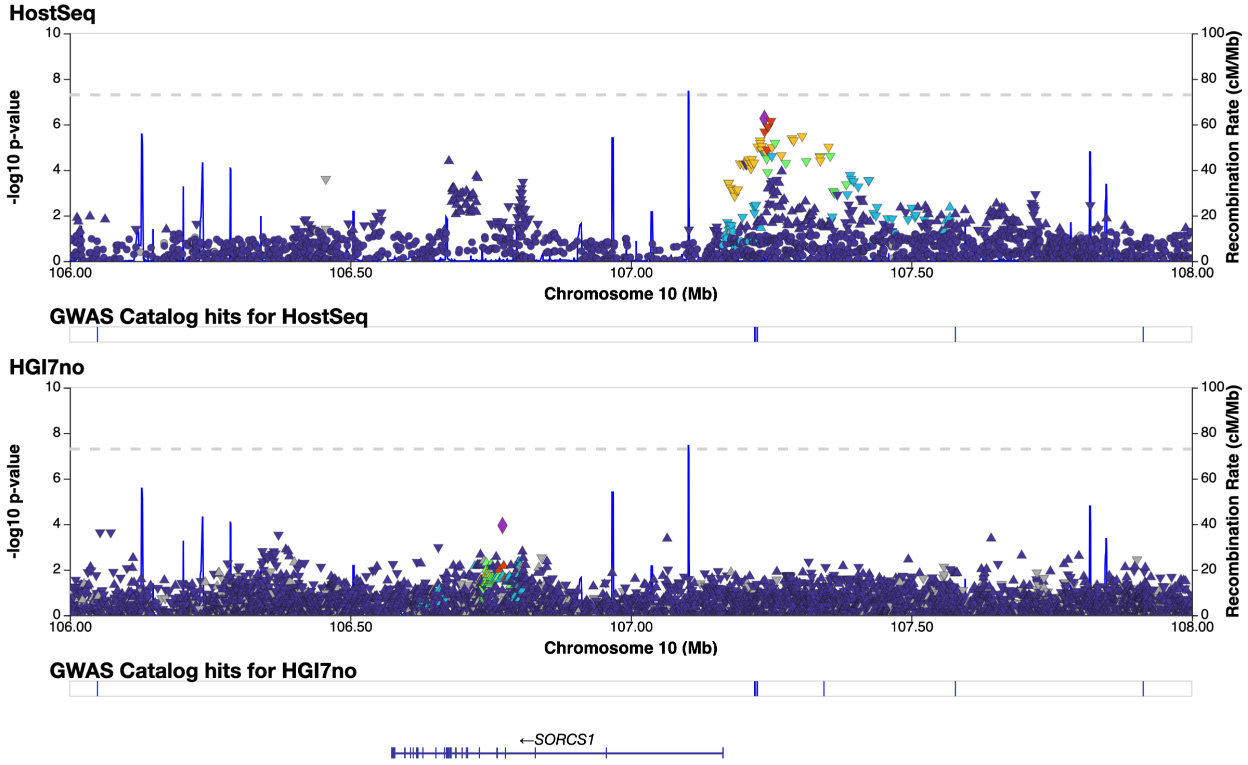
HostSeq
-log10 p-value
Recombination Rate (cM/Mb)
Chromosome 10 (Mb)
106.00
106.50
107.00
107.50
108.00
GWAS Catalog hits for HostSeq
HGI7no
GWAS Catalog hits for HGI7no
←SORCS1

c)

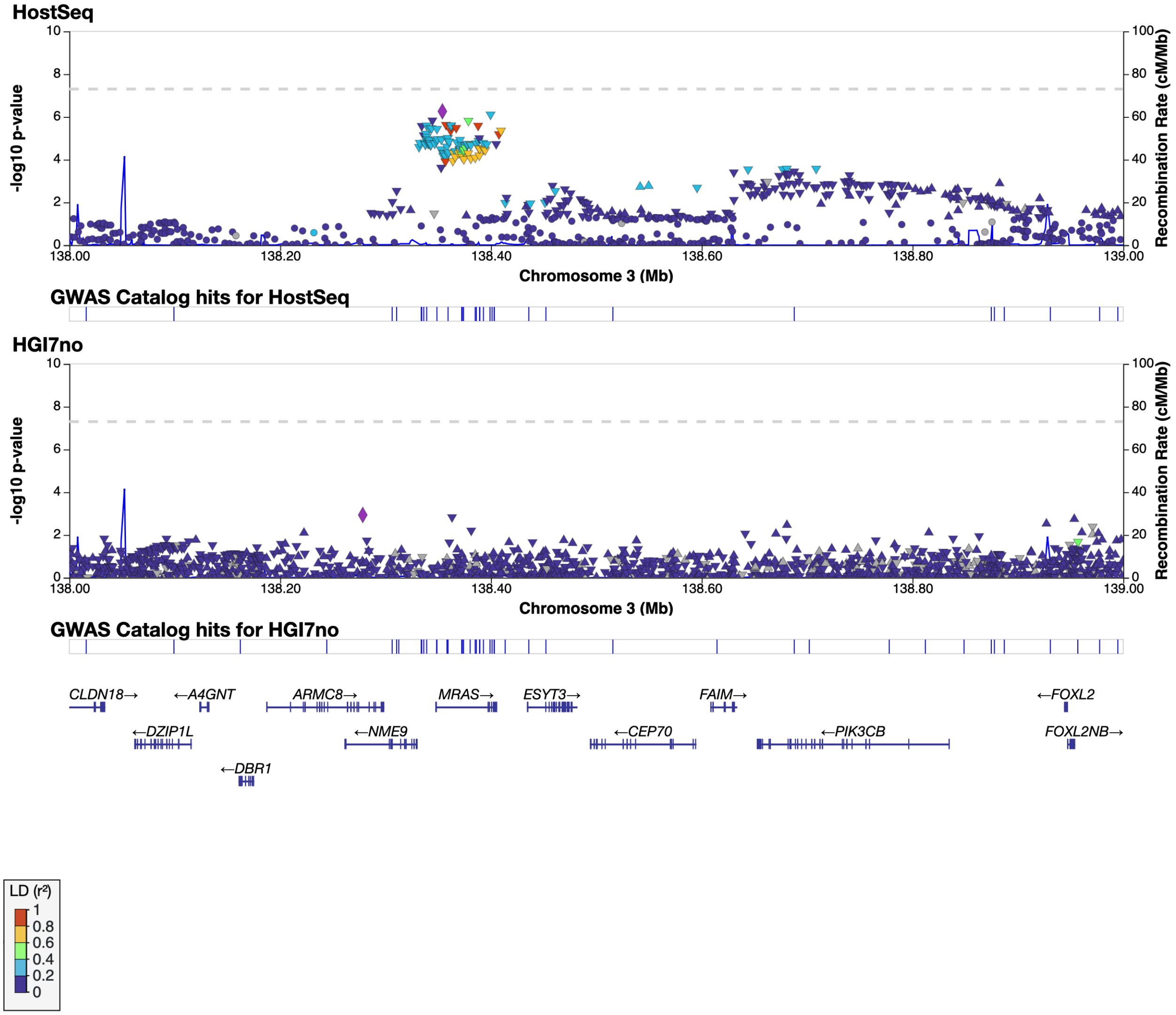

Figure S14. Region plots for the top three novel loci from HostSeq compared with HGI7no. Querying the three regions: a) chr15:54131608, b) chr10:107238146, c) chr3:138353967 in HostSeq (top row in each figure) with HGI7no (bottom row in each figure) shows that these variants are in LD with nearby variants. Plots were generated using myLocusZoom.
