# Supplementary material for: Canadian COVID-19 host genetics cohort replicates known severity associations
Source: PLoS Genet. 2024 Mar 22;20(3):e1011192. doi: 10.1371/journal.pgen.1011192 (PMC10990181; doi:10.1371/journal.pgen.1011192)
Supplement: S15 Fig — In the Manhattan plot, Y-axis indicates -Log10 p-values of MAGMA analysis for genes, X-axis indicates chromosomes. Grey horizontal line indicates Bonferroni significance level of P < 2.7E-6. In the corresponding QQ-plot, the X and Y axes indicate expected and observed -Log10 p-values, respectively (genomic control λ = 1.1). The significant hit on chromosome 3 is the MRAS gene with 91 SNPs and P = 3.52E-7. (PDF) [file pgen.1011192.s015.pdf]

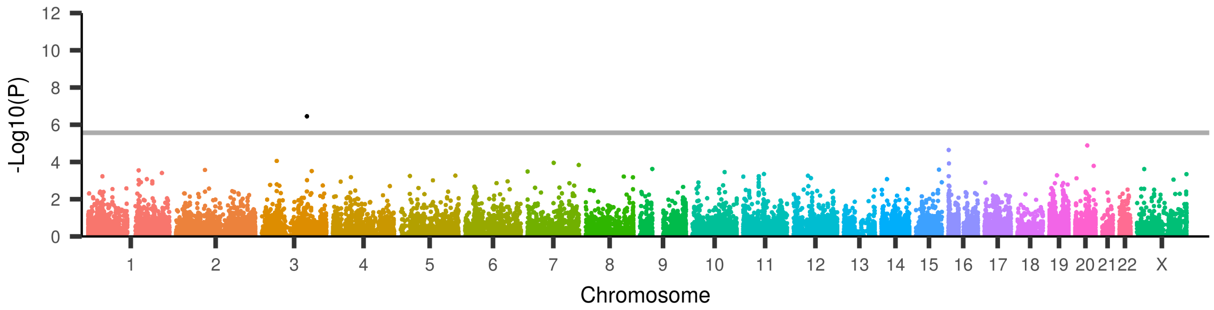

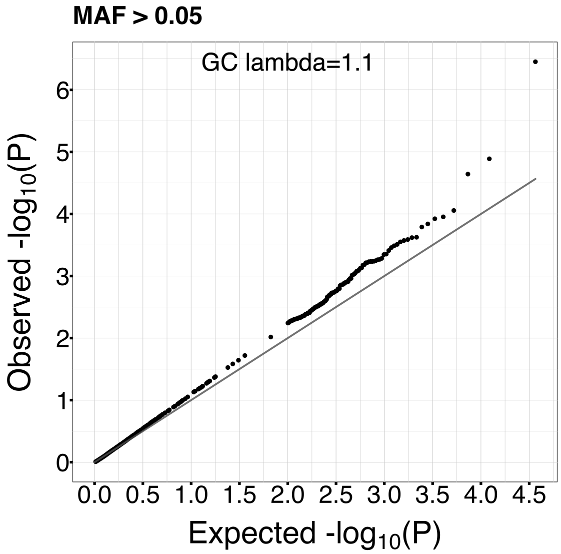

Figure S15. Gene-based test results of primary GWAS. Post-GWAS functional analysis of the primary HostSeq GWAS included a gene-based test computed by MAGMA. In the Manhattan plot, Y-axis indicates -Log10 p-values of MAGMA analysis for genes, X-axis indicates chromosomes. Grey horizontal line indicates Bonferroni significance level of $P < 2.7E\text{-}6$. In the corresponding QQ-plot, the X and Y axes indicate expected and observed -Log10 p-values, respectively (genomic control $\lambda = 1.1$). The significant hit on chromosome 3 is the *MRAS* gene with 91 SNPs and $P = 3.52E\text{-}7$.
