# Supplementary material for: Canadian COVID-19 host genetics cohort replicates known severity associations
Source: PLoS Genet. 2024 Mar 22;20(3):e1011192. doi: 10.1371/journal.pgen.1011192 (PMC10990181; doi:10.1371/journal.pgen.1011192)
Supplement: S16 Fig — SAIGE GWAS of five HostSeq ancestries (EAS, SAS, AFR, AMR and EUR) were meta-analyzed using MR-MEGA [N = 8,272]. In the Manhattan plot, Y-axis indicates -Log10 p-values of MR-MEGA analysis for variants with MAF > 5%, X-axis indicates chromosomes. Variants falling in the GIAB difficult-to-sequence regions have been excluded. Variants missing in any of the ancestry sets did not have a meta-analysis result. Grey horizontal line indicates genome-wide significance level of P < 5E-8. In the corresponding QQ-plot, the X and Y axes indicate expected and observed -Log10 p-values, respectively (genomic control λ = 0.991). (PDF) [file pgen.1011192.s016.pdf]

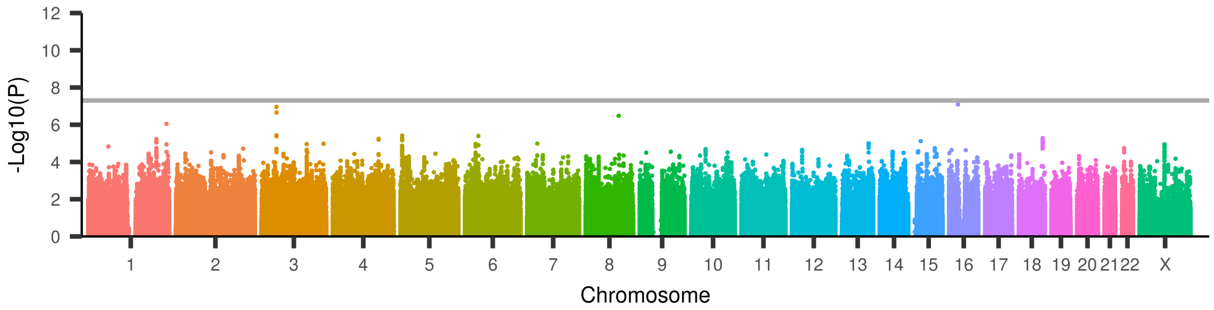

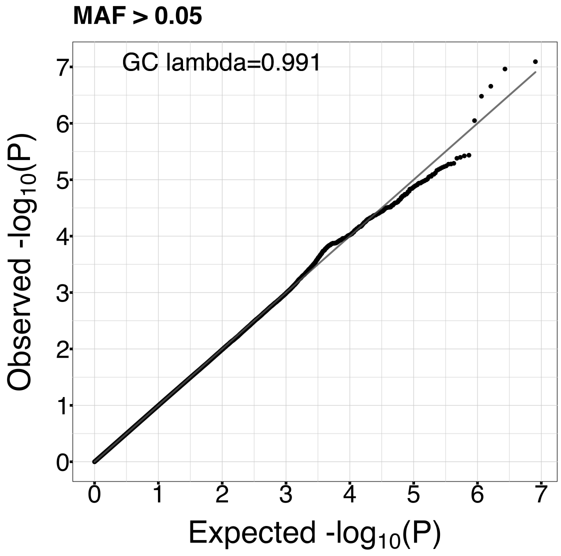

Figure S16. Meta-analysis results of ancestry-stratified GWAS. SAIGE GWAS of five HostSeq ancestries (EAS, SAS, AFR, AMR and EUR) were meta-analyzed using MR-MEGA [N = 8,272]. In the Manhattan plot, Y-axis indicates -Log10 p-values of MR-MEGA analysis for variants with MAF > 5%, X-axis indicates chromosomes. Variants falling in the GIAB difficult-to-sequence regions have been excluded. Variants missing in any of the ancestry sets did not have a meta-analysis result. Grey horizontal line indicates genome-wide significance level of P < 5E-8. In the corresponding QQ-plot, the X and Y axes indicate expected and observed -Log10 p-values, respectively (genomic control $\lambda = 0.991$).
