# Supplementary material for: Canadian COVID-19 host genetics cohort replicates known severity associations
Source: PLoS Genet. 2024 Mar 22;20(3):e1011192. doi: 10.1371/journal.pgen.1011192 (PMC10990181; doi:10.1371/journal.pgen.1011192)
Supplement: S17 Fig — The p-values are derived from a 2 degrees-of-freedom test that considers both genotype, and interaction between genotype and sex jointly. In the Manhattan plot, Y-axis indicates -Log10 p-values of regenie analysis for variants with MAF > 5%, X-axis indicates chromosomes. Variants falling in the GIAB difficult-to-sequence regions have been excluded. Grey horizontal line indicates genome-wide significance level of P < 5E-8. In the corresponding QQ-plot, the X and Y axes indicate expected and observed -Log10 p-values, respectively (genomic control λ = 1.194). (PDF) [file pgen.1011192.s017.pdf]

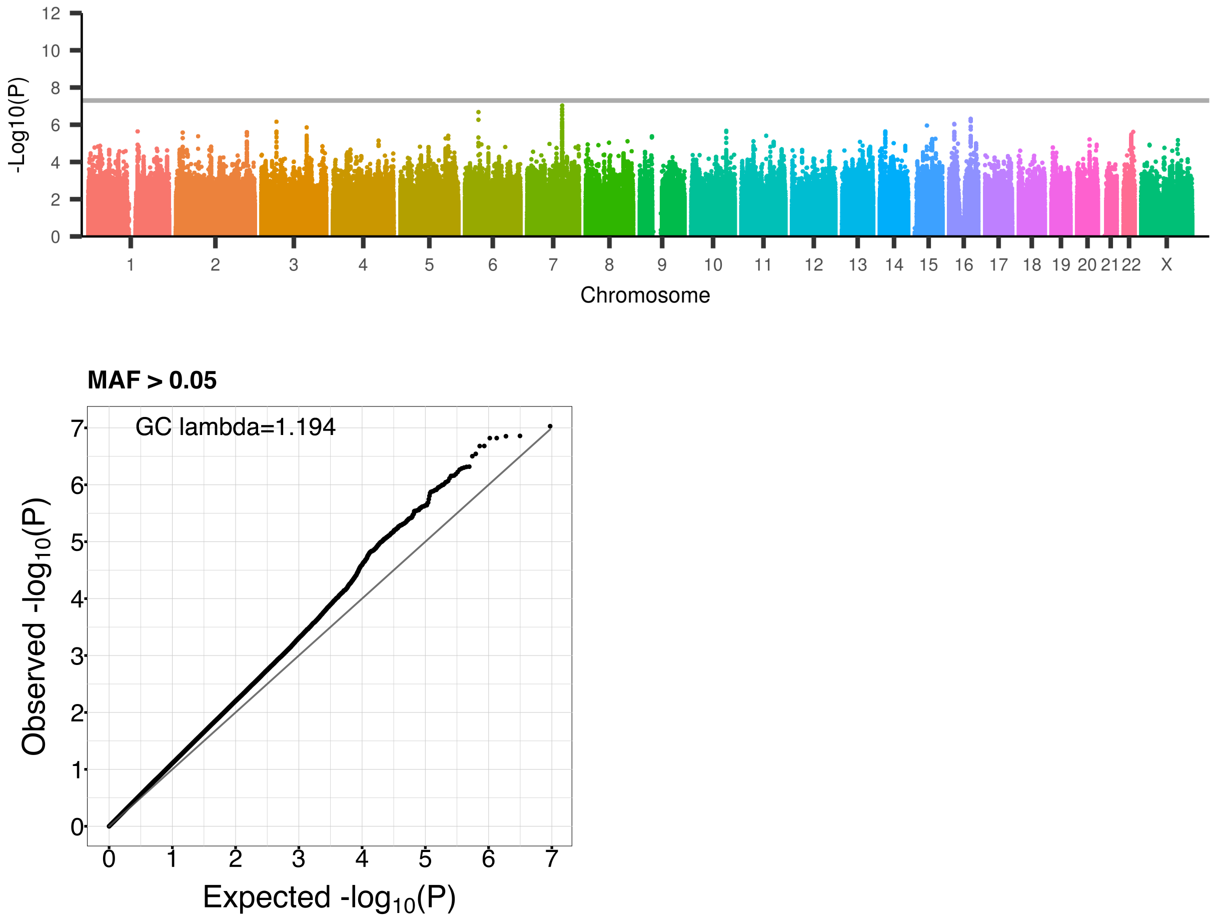

Figure S17. GWAS testing the G x Sex interaction effect. The p-values are derived from a 2 degrees-of-freedom test that considers both genotype, and interaction between genotype and sex jointly. In the Manhattan plot, Y-axis indicates -Log10 p-values of regenie analysis for variants with MAF $>$ 5%, X-axis indicates chromosomes. Variants falling in the GIAB difficult-to-sequence regions have been excluded. Grey horizontal line indicates genome-wide significance level of P $<$ 5E-8. In the corresponding QQ-plot, the X and Y axes indicate expected and observed -Log10 p-values, respectively (genomic control $\lambda = 1.194$).
