# Supplementary material for: Canadian COVID-19 host genetics cohort replicates known severity associations
Source: PLoS Genet. 2024 Mar 22;20(3):e1011192. doi: 10.1371/journal.pgen.1011192 (PMC10990181; doi:10.1371/journal.pgen.1011192)
Supplement: S18 Fig — Querying the chr7:107127037 region in G x Sex GWAS (top row) shows that this variant is in LD with nearby variants. Comparing it with the following in order: primary GWAS, sex-stratified GWAS for males, sex-stratified GWAS for females, and HGI7no shows that there is a sex-effect for this locus in HostSeq, where males [N = 3,646] have an association with hospitalization. Plots were generated using myLocusZoom. (PDF) [file pgen.1011192.s018.pdf]

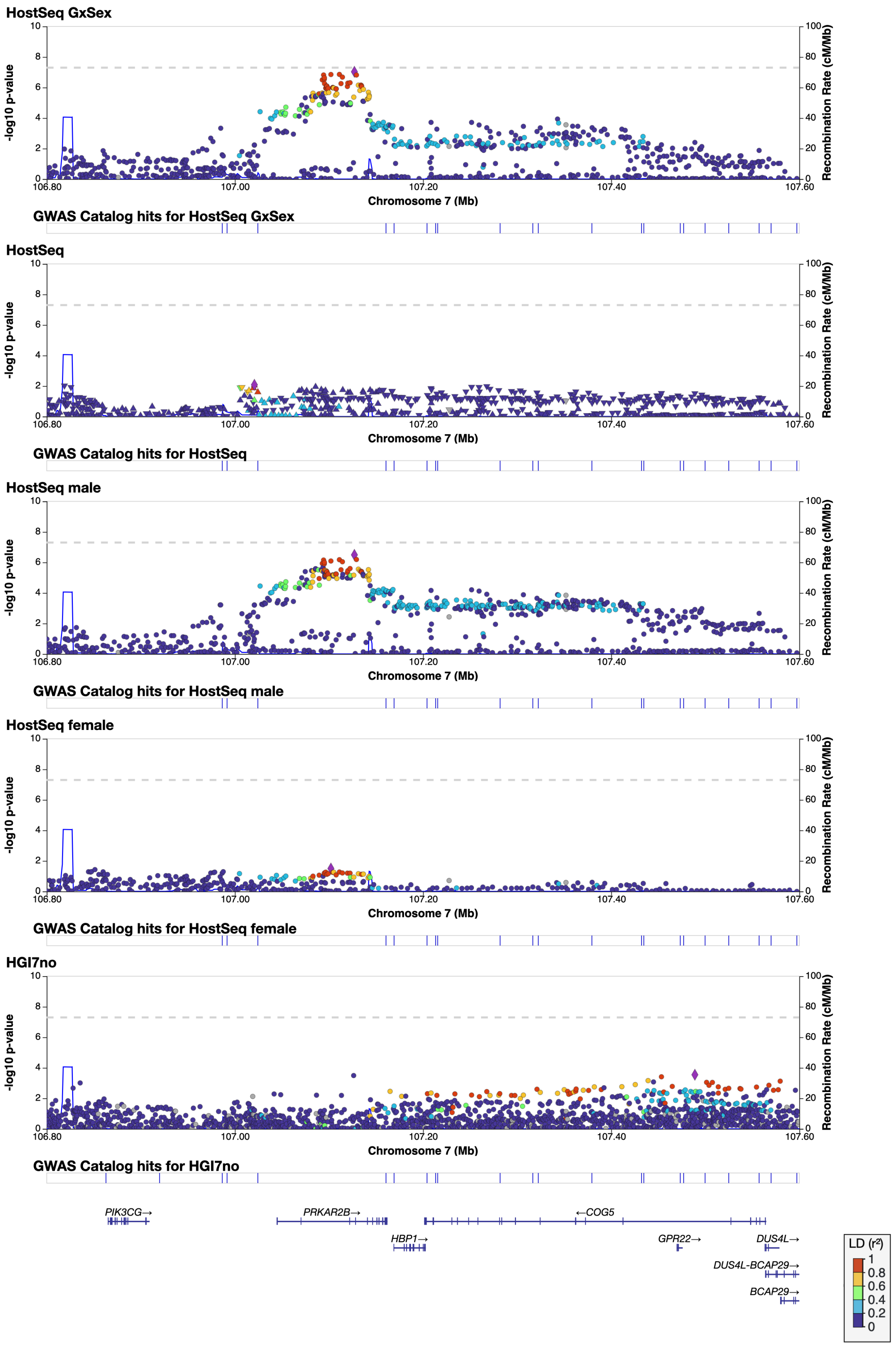
HostSeq GxSex
-log10 p-value
Recombination Rate (cM/Mb)
Chromosome 7 (Mb)
GWAS Catalog hits for HostSeq GxSex
HostSeq
GWAS Catalog hits for HostSeq
HostSeq male
GWAS Catalog hits for HostSeq male
HostSeq female
GWAS Catalog hits for HostSeq female
HGI7no
GWAS Catalog hits for HGI7no
PIK3CG→
PRKAR2B→
←COG5
HBP1→
GPR22→
DUS4L→
DUS4L-BCAP29→
BCAP29→
LD (r²)

Figure S18. Region plot for the top novel locus identified through the G x Sex interaction test compared with other results. Querying the chr7:107127037 region in G x Sex GWAS (top row) shows that this variant is in LD with nearby variants. Comparing it with the following in order: primary GWAS, sex-stratified GWAS for males, sex-stratified GWAS for females, and HGI7no shows that there is a sex-effect for this locus in HostSeq, where males [N = 3,646] have an association with hospitalization. Plots were generated using myLocusZoom.
