# Supplementary material for: Canadian COVID-19 host genetics cohort replicates known severity associations
Source: PLoS Genet. 2024 Mar 22;20(3):e1011192. doi: 10.1371/journal.pgen.1011192 (PMC10990181; doi:10.1371/journal.pgen.1011192)
Supplement: S19 Fig — Top) Manhattan plot for the high impact set (3,350 genes). Middle) Manhattan plot for the high/moderate impact set (17,341 genes). Bottom) QQ-plots for the high impact (bottom left), and high/moderate impact (bottom right) sets. SKAT-O analysis was performed on variants outside the GIAB difficult-to-sequence regions. (PDF) [file pgen.1011192.s019.pdf]

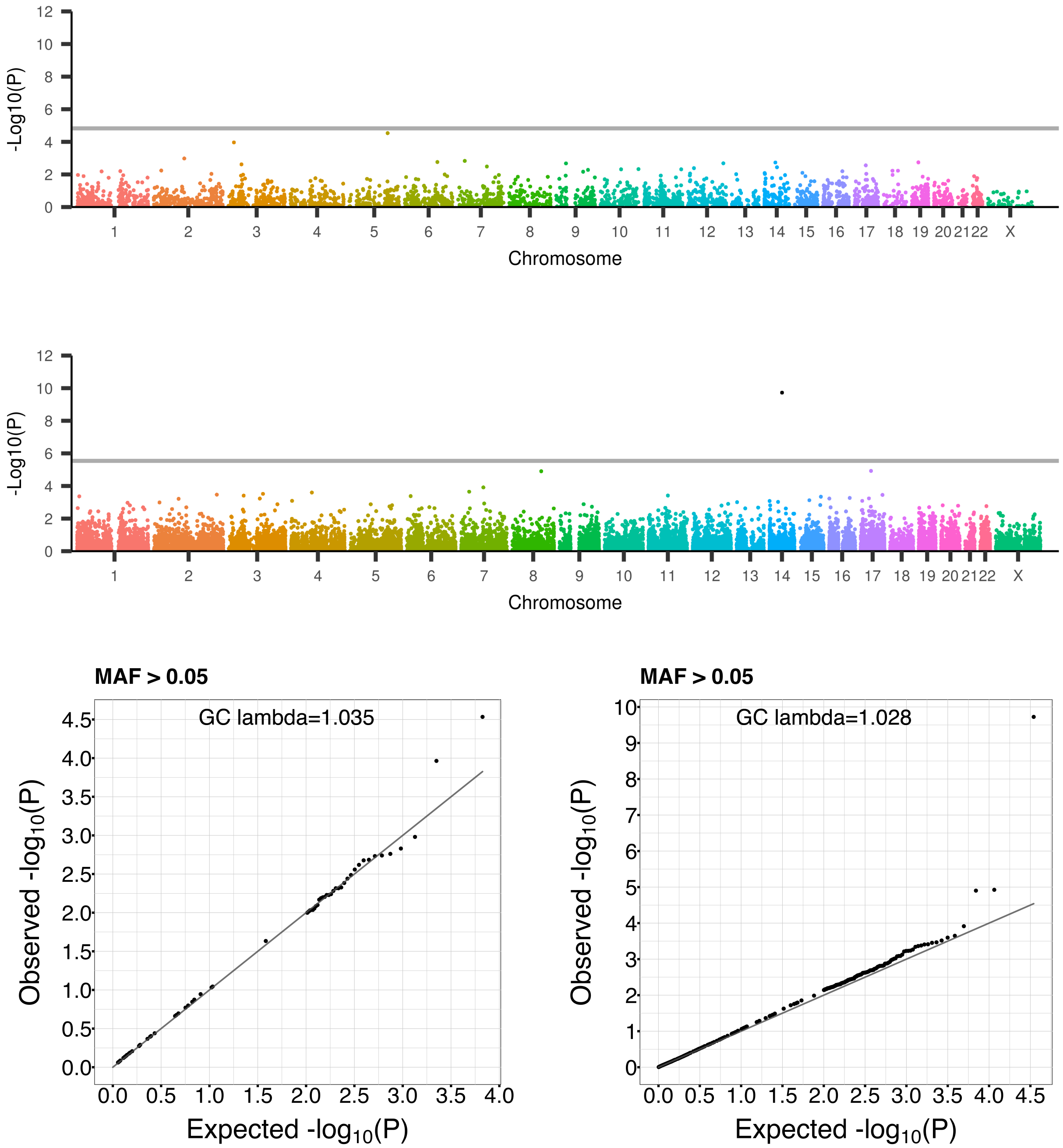

Figure S19. SKAT-O results for gene-based testing including rare variants. Top) Manhattan plot for the high impact set (3,350 genes). Middle) Manhattan plot for the high/moderate impact set (17,341 genes). Bottom) QQ-plots for the high impact (bottom left), and high/moderate impact (bottom right) sets. SKAT-O analysis was performed on variants outside the GIAB difficult-to-sequence regions.
